# ZIF-67 functionalized bacterial cellulose films for light-enhanced NaBH_4_-assisted catalytic reduction of nitrobenzene

**DOI:** 10.1039/d6ra04058f

**Published:** 2026-09-21

**Authors:** Zandile Mhlwatika, Samson Khene, Nolwazi Nombona

**Affiliations:** a Department of Chemistry, University of Pretoria Private Bag X20 Hatfield 0028 South Africa nolwazi.nombona@up.ac.za; b University College London (UCL), Department of Chemistry Christopher Ingold Building, 20 Gordon Street London WC1H 0AJ UK

## Abstract

This study reports on the synthesis of ZIF-67 and its *in situ* growth on bacterial cellulose (BC) films produced through a green fermentation method. The resulting BCZIF-67 composites were characterized by N_2_ sorption analysis, FTIR, XRD, SEM-EDS, and UV-vis diffuse reflectance spectroscopy. The results supported the successful incorporation of ZIF-67 into the BC matrix, with well-dispersed ZIF-67 particles distributed throughout the porous BC network. Under optimized catalytic conditions (6 mg catalyst, 600 mM NaBH_4_, 1 mM nitrobenzene), BCZIF-67 achieved 100% nitrobenzene reduction efficiency within 13 min under UV irradiation (*λ* = 365 nm), compared to 40% for pristine ZIF-67 under identical conditions. Band-edge estimates and scavenger experiments were consistent with synergistic contributions from photoinduced charge-transfer processes and NaBH_4_ derived reducing equivalents. BCZIF-67 maintained high catalytic activity during the first four reuse cycles, retaining a reduction efficiency of 90% in the fourth cycle. The efficiency subsequently decreased to 45% in the fifth cycle with confirmed cobalt leaching, demonstrating the need to improve the long-term stability of the composite. The incorporation of renewable bacterial cellulose offers a sustainable, bio-based strategy for designing ZIF-cellulose catalysts for environmental pollutant remediation.

## Abstract 2

I am providing an improved, higher-quality version of the image to enhance its clarity, readability and overall presentation. The scientific content remains unchanged.

## Graphical abstract

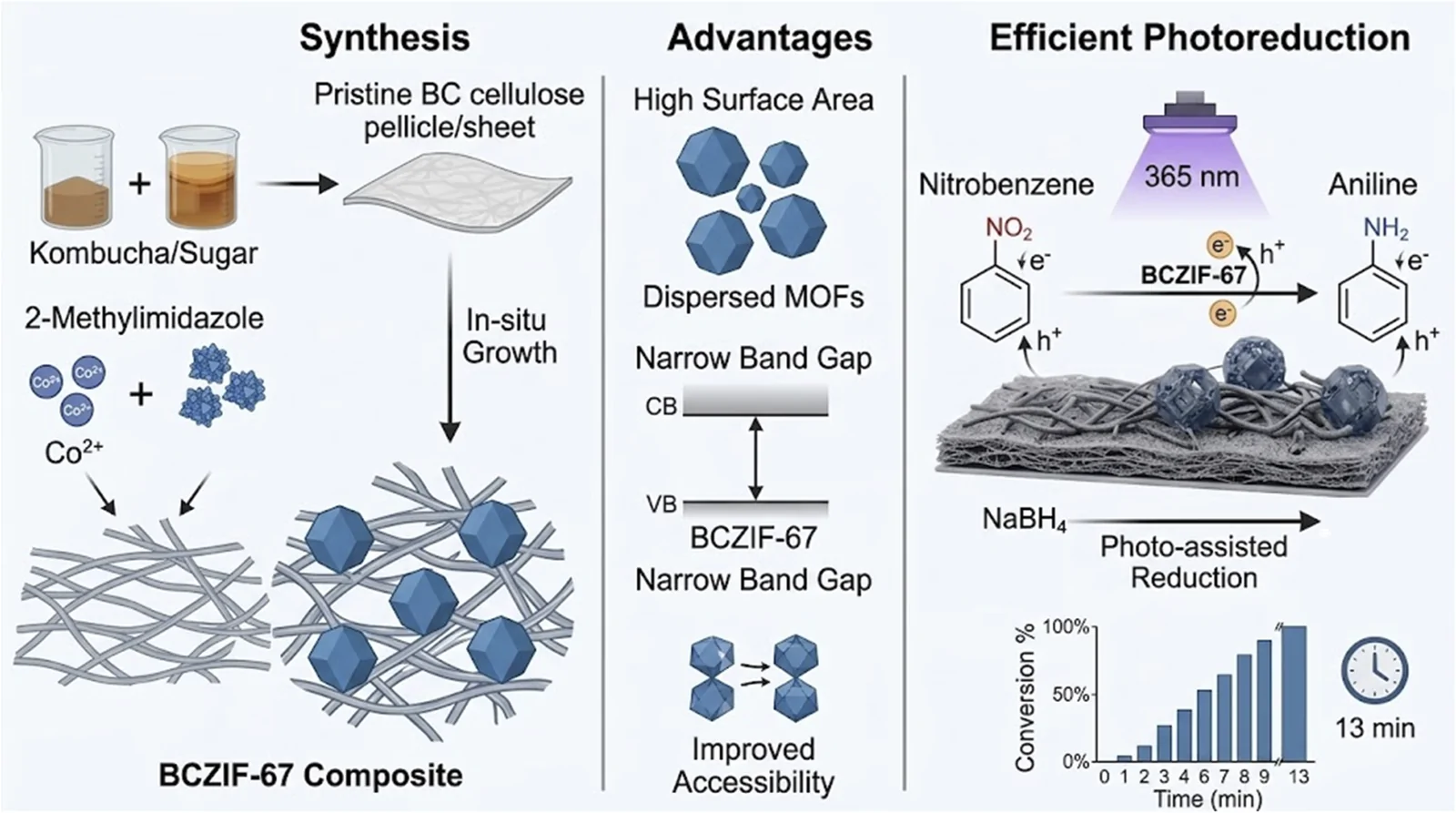

## 1. Introduction

Water contamination remains a pressing global challenge, exacerbated by rapid industrialization and intensive agricultural practices that release dyes, heavy metals, and nitroaromatic compounds into aquatic environments.^1,2^ Among these pollutants, nitrobenzene (NB) is of particular concern because of its widespread use as an industrial chemical intermediate in the manufacture of dyes, explosives, cosmetics, and pesticides.^3^ Substantial quantities of NB enter water systems annually, where its strong electron-withdrawing nitro (–NO_2_) group confers chemical stability and resistance to biodegradation, resulting in the persistence of NB in contaminated water.^4,5^ Given its persistence and toxicity, several regulatory agencies have established strict guidelines for NB concentrations in environmental matrices. In South Africa, the Department of Environmental Affairs established land use-specific soil screening values for NB, including 2.8 mg kg^−1^ for all land uses and informal residential areas and 2.9 mg kg^−1^ for standard residential areas.^6^ Although the United States Environmental Protection Agency (EPA) has not established a federal maximum contaminant level (MCL) for NB, state-level limits are enforced.^7^ The New York State Department of Health applies a drinking water MCL of 5 µg L^−1^ under its principal organic contaminant framework. In addition, New York State has established a more stringent ambient water quality value of 0.4 µg L^−1^ to protect sources of potable water.^8^ These regulatory benchmarks highlight the need for rigorous monitoring and control of NB to reduce its environmental and public health impacts.

Catalytic reduction of NB to aniline (AN) provides a valuable remediation pathway because AN is less toxic and more biodegradable^9^ and serves as an industrial precursor for antioxidants, dyes, and plastics.^10–12^ Although noble metal catalysts such as platinum (Pt), palladium (Pd), and ruthenium (Ru) have shown high efficiency for this transformation, their high cost and limited availability hinder their large-scale application.^13^ For example, a Pd/C catalyst can cost between $232 and $680 per gram,^14^ while Pt-based catalysts typically range from $129 to $298 per gram.^15^ These high costs make such materials economically impractical for wastewater treatment, and their scalability remains limited.^16^ In contrast, metal–organic frameworks (MOFs), particularly zeolitic imidazolate frameworks (ZIFs), offer a cost-effective and scalable alternative.^17^ MOFs can be synthesized under mild conditions from relatively abundant precursors and are characterized by high surface areas, tunable porosity, and versatile chemistry.^18^ Among them, ZIF-67 can be produced at a comparatively low cost, ranging from $75 to $80 per gram, with moderate scalability.^19^ These economic and scalability advantages contrast sharply with those of noble metal catalysts, whose high costs and limited availability hinder large-scale application, positioning MOFs as potentially more practical and sustainable candidates for industrial-scale NB remediation.

While MOFs offer clear advantages, their practical application is limited by intrinsic drawbacks such as particle aggregation and rapid electron–hole recombination, which limit catalytic performance.^20^ To address these limitations, recent strategies have focused on integrating MOFs with high surface area supports to improve stability, dispersion, and accessibility of active sites.^21^ In this context, BC represents a suitable support material. It is renewable, biodegradable, and characterized by high mechanical strength, hydrophilicity, and an abundance of surface functional groups.^22^ These features enable strong interactions with MOFs, enhancing their dispersion and catalytic accessibility. Nevertheless, BC is an insulating material that can swell or degrade under photocatalytic conditions, and may scatter light or hinder substrate diffusion, which can limit overall efficiency.

Previous studies have already demonstrated the synergistic benefit of cellulose-MOF composites for adsorption and catalysis.^23^ To the best of our knowledge, this is the first study to investigate the light-enhanced, NaBH_4_-assisted catalytic reduction of NB using a MOF-cellulose composite, addressing a clear research gap despite the recognised potential of these materials in adsorption and catalysis. In this work, we report a simple strategy for preparing ZIF-67-embedded BC films, to enhance light-assisted catalytic performance. Incorporating ZIF-67 into the BC matrix was designed to improve structural stability and active site accessibility, while mitigating intrinsic drawbacks of pristine ZIF-67 such as rapid electron–hole recombination and particle aggregation. This approach establishes a promising platform for the design of more sustainable catalytic materials for environmental remediation.

## 2 Materials and methods

### 2.1 Materials

Potassium dihydrogen phosphate (KH_2_PO_4_, 99.99%); magnesium sulfate heptahydrate (MgSO_4_·7H_2_O, 99.99%); ammonium sulfate ((NH_4_)_2_SO_4_, 99.0%); potassium iodide (KI, 99.0%); sodium hydroxide (NaOH, 97.0%); sodium borohydride (NaBH_4_, 99.0%); sodium hypochlorite (NaClO, 15%); sucrose (C_12_H_22_O_11_, 99.5%); cobalt nitrate hexahydrate (Co(NO_3_)_2_·6H_2_O, 98.0%); 2-methylimidazole (C_4_H_6_N_2_, 99.0%); NB (C_6_H_5_NO_2_, 99.0%); *N*,*N*-dimethylformamide (HCON(CH_3_)_2_, 99.8%); glacial acetic acid (CH_3_COOH, 99.8%); ascorbic acid (C_6_H_8_O_6_, 99.0%); and ethanol (CH_3_CH_2_OH, 99.5%) were used. All chemicals were obtained from Sigma-Aldrich (South Africa) and used as received. Kombucha tea, yeast, green tea, and brown sugar were purchased from commercial retailers in South Africa. Deionized water was used throughout the study.

### 2.2 Biosynthesis of bacterial cellulose (BC)

Green tea bags were steeped in 1000 mL of boiling water, after which 80 g of brown sugar was added, and the mixture was stirred at 350 rpm for 1 hour. The tea mixture was cooled to room temperature and then mixed with 1000 mL of kombucha. The initial pH of the mixture was 2.7. The resulting mixture was transferred to sterilized beakers, covered with semi-permeable cloths, and incubated statically under ambient air in the dark at room temperature. Visible growth of a symbiotic culture of bacteria and yeast (SCOBY) was observed at the liquid–air interface after 3 days. The SCOBY was recovered after 14 days and used to initiate BC biosynthesis.

BC was biosynthesized according to a previously reported procedure.^24^ A Hestrin-Schramm (HS) medium was prepared by dissolving sucrose (50.0 g), potassium dihydrogen phosphate (3.0 g), ammonium sulfate (5.0 g), magnesium sulfate heptahydrate (0.05 g), and yeast (5.0 g) in 1000 mL of boiling water. The HS medium was cooled to room temperature before the SCOBY was introduced. The resulting mixture was covered with semi-permeable cloth and incubated under static conditions in the dark at room temperature. After 14 days, the BC pellicle was recovered and washed with water. The BC pellicle was then treated with 0.25 M NaOH for 24 hours, followed by bleaching with NaClO solution (15 wt%) for 4 hours. The bleached BC pellicle was thoroughly washed with water and stored in distilled water.

### 2.3 Synthesis of BC-supported ZIF-67 (BCZIF-67)

BCZIF-67 was synthesized using a previously reported procedure with minor modifications.^25^ Briefly, 291.05 mg of cobalt nitrate hexahydrate was dissolved in a mixture of 15 mL of DMF and 1 mL of acetic acid. A BC pellicle (approximately 1.5 × 1.5 cm) was immersed in the cobalt precursor solution and stirred at 350 rpm at room temperature for 5 hours to promote adsorption of cobalt ions onto the BC surface. Separately, 492.6 mg of 2-methylimidazole was dissolved in a mixture of 15 mL of DMF and 1 mL of acetic acid. The cobalt containing BC suspension and the 2-methylimidazole solution were then combined, and the resulting reaction mixture was stirred at 350 rpm at 80 °C for 24 hours under ambient air. After heating, the mixture was allowed to cool to room temperature and was aged at room temperature for an additional 24 hours to complete the *in situ* formation of ZIF-67 within the BC matrix. BC provides a suitable support for the *in situ* growth of ZIF-67 because of the abundance of hydroxyl groups on its surface. These functional groups can coordinate with Co^2+^ ions, anchoring them to cellulose fibrils and creating localized nucleation sites. Subsequent interactions between the anchored Co^2+^ ions and the 2-methylimidazolate linkers promote heterogeneous nucleation and growth of ZIF-67 directly on the BC network rather than in the bulk solution. This process favors the dispersion of ZIF-67 crystals throughout the porous BC matrix and the formation of a stable composite with accessible active sites.^26^ The resulting BCZIF-67 composite was carefully collected, washed several times with ethanol, and dried in an oven at 40 °C for 12 hours. For comparison, pristine ZIF-67 powder was prepared under the same conditions in the absence of BC.

### 2.4 Characterization techniques

Fourier transform infrared (FTIR) spectra were collected using a Bruker Alpha FTIR spectrometer equipped with a platinum attenuated total reflectance (ATR) accessory. Spectra were recorded over the range 4000–400 cm^−1^ at a resolution of 4 cm^−1^. Prior to each measurement, the ATR crystal was cleaned with acetone, and a background spectrum was collected. X-ray diffraction (XRD) analysis was performed using a Bruker D2 PHASER-e diffractometer with Cu-Kα radiation (*λ* = 0.15418 nm). Diffraction patterns were collected over a 2*θ* range of 5–50°, using a step size of 0.05° and a scan speed of 5 steps per s. Thermogravimetric analysis (TGA) was performed using an SDT Q600 V20.9 Build 20 Module DSC-TGA instrument. Approximately 5 mg of each sample was analyzed in an aluminum crucible over the temperature range 20–800 °C at a heating rate of 20 °C min^−1^ under a nitrogen flow of 15 mL min^−1^. The Brunauer–Emmett–Teller (BET) surface area and Barrett–Joyner–Halenda (BJH) pore size distribution were determined using an Autosorb iQ analyzer (model 7, ASiQWin version 5.2x), with N_2_ as the adsorbate gas. Before analysis the samples were degassed at 120 °C for 12 hours. Nitrogen adsorption–desorption isotherms were recorded at 77 K over a relative-pressure (*P*/*P*_o_) range of 0–1.0.

Surface morphology and elemental composition were examined using a Zeiss Crossbeam 540 field-emission scanning electron microscope (FEG-SEM) equipped with energy-dispersive X-ray spectroscopy (EDS). Samples were mounted on SEM stubs, and carbon-coated using a Quorum Q150T ES sputter coater and analyzed at an accelerating voltage of 2 kV. Secondary electron images were acquired for morphological analysis. Ultraviolet-visible (UV-vis) absorption measurements were carried out using a CARY 100 BIO UV-vis spectrophotometer over the wavelength range 200–800 nm. Cobalt (Co) leaching from the BCZIF-67 photocatalyst during the recycling experiments was quantified using an Agilent 4100 microwave plasma atomic emission spectrometer (MP-AES) equipped with a multimode sample introduction system (MSIS), a concentric glass nebulizer, a standard torch, and an external gas control module.

### 2.5 NaBH_4_-assisted photocatalytic reduction of NB

The photocatalytic reduction of NB was performed using ZIF-67 and BCZIF-67 as photocatalysts, with NaBH_4_ as the reducing agent. The procedure was adapted from literature with minor modifications.^27^

Initial optimization experiments were conducted by varying one parameter at a time. Based on these experiments and unless otherwise stated, all subsequent experiments were conducted under the optimized conditions: 6 mg of ZIF-67 or BCZIF-67 was added to 100 mL of 1 mM NB solution containing 600 mM NaBH_4_. The reaction mixture was stirred at 350 rpm at room temperature for 10 minutes in the dark. The mixture was subsequently irradiated with ultraviolet light (*λ* = 365 nm) under ambient atmospheric pressure. At one-minute intervals, 0.1 mL aliquots were withdrawn from the reaction mixture. Each aliquot was then diluted with 3 mL of ethanol and analyzed by UV-vis spectroscopy. The kinetic data were fitted to zero-, first-, and second-order models to obtain the corresponding apparent absorbance-based rate constants.

### 2.6 Scavenger addition experiments

Scavenger-addition experiments were conducted to evaluate the effects of potassium iodide and ascorbic acid on the UV-irradiated reduction of NB. Potassium iodide and ascorbic acid were tested separately as redox-active additives; however, because these reagents are not fully selective, the results were interpreted qualitatively rather than used to identify individual reactive species. A total of 6 mg of catalyst was added to a 1 mM NB solution containing 600 mM NaBH_4_, followed by the addition of 1 mM of a trapping agent. All other reaction conditions were identical to those used for the standard photocatalytic reduction experiment, and the reduction efficiency was determined after 3 min of UV irradiation.

## 3 Results and discussion

### 3.1 Fourier-transform infrared spectroscopy (FTIR)

FTIR spectroscopy was used to identify functional groups present in the synthesised materials. The spectrum of ZIF-67 (Fig. 1a) exhibited characteristic bands in the range 1603–750 cm^−1^, which are attributed to the stretching and bending vibrations of the imidazole ring. These bands are consistent with the framework formed by 2-methylimidazole linkers coordinated to cobalt ions.^28^ A band at 431 cm^−1^ corresponds to the Co–N stretching vibration, supporting the successful formation of the metal–imidazolate coordination framework characteristic of ZIF-67. These results are consistent with previous reports.^29^

**Fig. 1:**
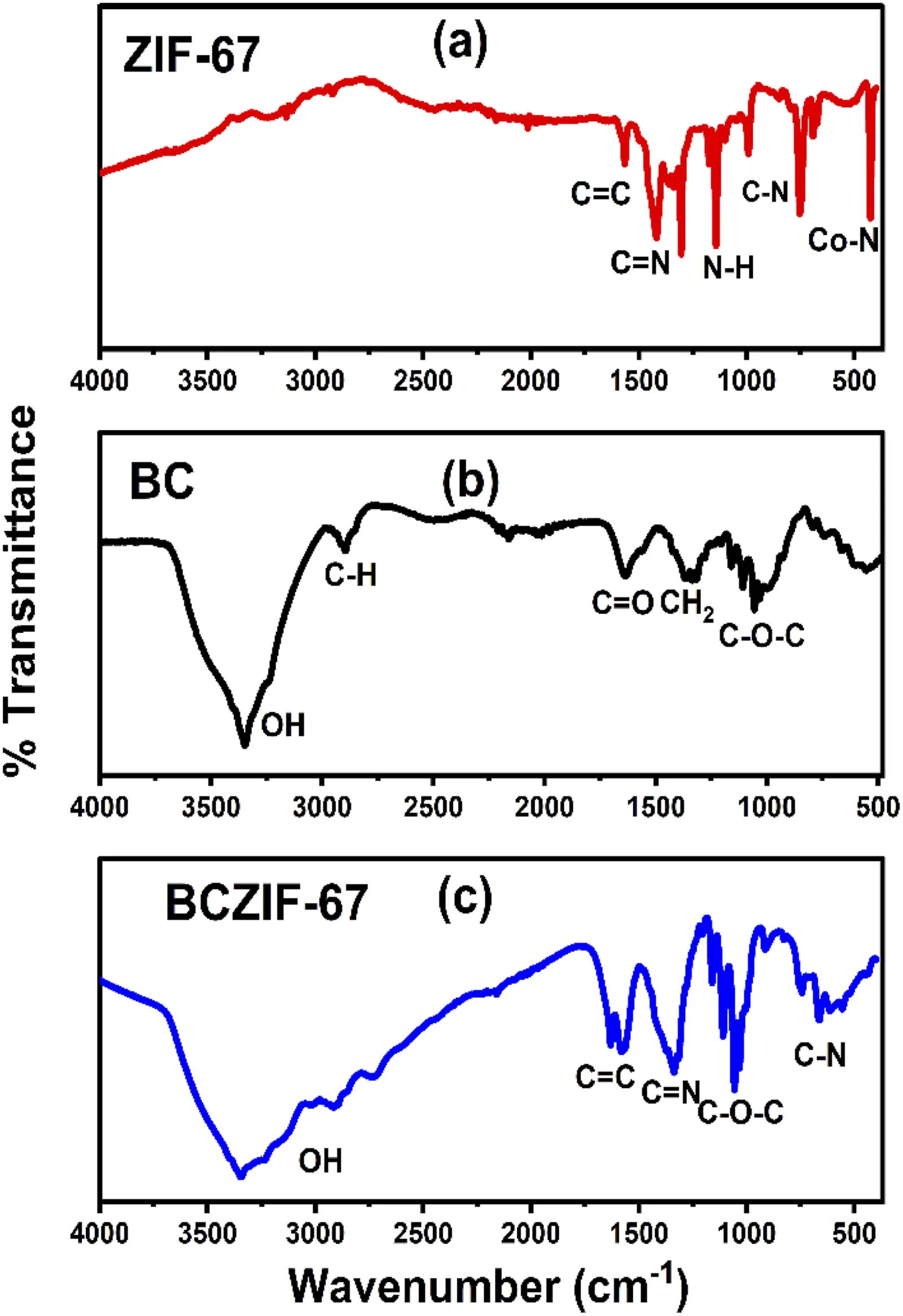
FTIR spectra of (a) ZIF-67, (b) BC, and (c) BCZIF-67.

The BC spectrum (Fig. 1b) displayed a broad band at 3400 cm^−1^, assigned to O–H stretching vibrations associated with intra-and intermolecular hydrogen bonding in cellulose. The peak at 2930 cm^−1^ corresponds to C–H stretching vibrations, while the peak at 1670 cm^−1^ is attributed to the bending vibration of absorbed water. The peak at 1400 cm^−1^ corresponds to CH_2_ bending vibrations within the β-d-glucopyranose units. The peaks between 1200–1050 cm^−1^ are ascribed to C–O–C stretching vibrations of β-1,4-glycosidic linkages, consistent with the formation of the polysaccharide backbone structure of BC.^30^ For the BCZIF-67 composite (Fig. 1c), the retention of the broad OH peak at 3400 cm^−1^ indicates hydroxyl binding sites remain available even after ZIF-67 integration. Concurrently, the imidazolate-associated peaks in the 1600–650 cm^−1^ region support the *in situ* growth of ZIF-67 on the BC matrix. Additionally, the peak at 1050 cm^−1^ is attributed to the C–O–C stretching vibration of β-1,4-glycosidic linkages, further supporting the structural integrity of the BC backbone within the composite.

### 3.2 X-ray diffraction (XRD)

XRD was used to examine the crystalline structure and phase purity of the synthesized materials. ZIF-67 (Fig. 2a) showed diffraction peaks at 2*θ* = 7.4°, 10.4°, 12.7°, 17.9°, and 24.4°. These peaks were consistent with the literature, confirming the characteristic crystal structure of ZIF-67.^31–33^ The XRD pattern of BC showed peaks at 2*θ* = 16.9° and 22.9°, as shown in Fig. 2b. These diffraction peaks agree well with reported values and are characteristic of crystalline BC.^34,35^ The diffractogram of BCZIF-67 (Fig. 2c) showed ZIF-67 peaks at 2*θ* = 7.5°, 10.5°, and 12.5°, indicating that the structural integrity of ZIF-67 was retained after incorporation into BC. Additionally, the diffraction peaks at 2*θ* = 14.5°, 16.7°, and 22.9° support the retention of the crystalline structure of BC within the BCZIF-67 composite. The average crystallite size of the synthesized materials was determined using the Scherrer equation (eqn (1)),1
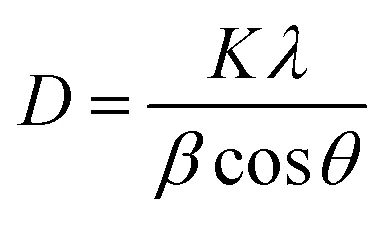
where *D* denotes the average crystallite size (nm), *K* is a dimensionless factor with a value of 0.94, *λ* is the X-ray wavelength (*λ* = 0.15418 nm), *β* is the full width at half maximum (FWHM) in radians, and *θ* is the Bragg angle in radians. The calculated average crystallite sizes ranged from 19.26 to 63.36 nm, as shown in Table 1. Interestingly, supporting ZIF-67 with BC resulted in a marked reduction in crystallite sizes. The smaller crystallite size may increase the fraction of exposed active sites and may facilitate improved interfacial charge transfer during photocatalysis.^36^ This marked decrease may increase the availability of surface sites and thereby contribute to the improved catalytic performance of BCZIF-67 during NB photoreduction.

**Fig. 2:**
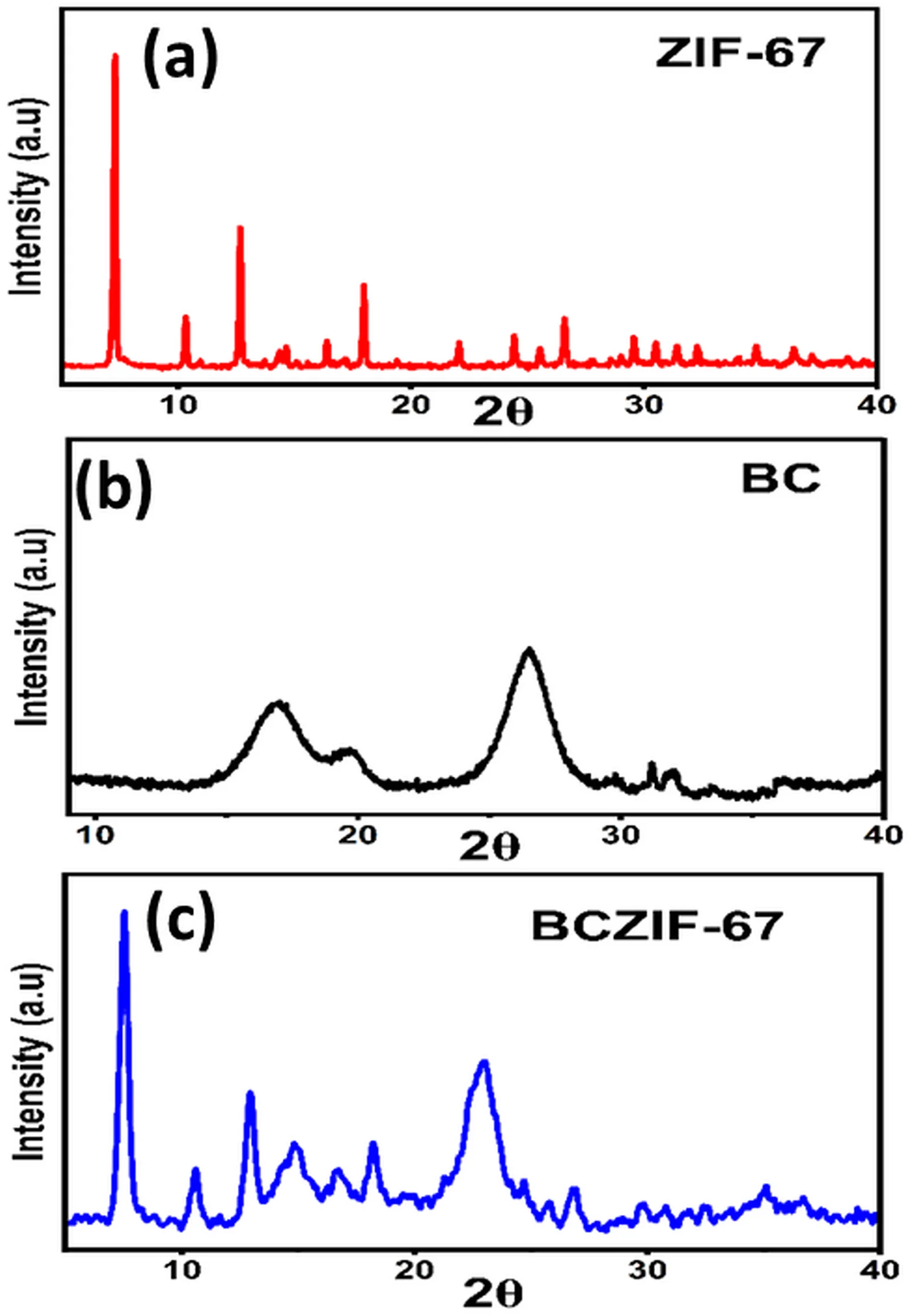
XRD patterns of (a) ZIF-67, (b) BC, and (c) BCZIF-67.

**Table 1:** Specific surface area, pore volume, pore diameter, and crystallite size of the synthesized materials

Sample	*S*_BET_ (m^2^ g^−1^)	Pore volume (cm^3^ g^−1^)	Pore diameter (nm)	Average crystallite size (nm)^a^	References
ZIF-67	1656	0.46	0.830	63.36	This work
BC	15.09	0.060	5.88	26.68	
BCZIF-67	1305	0.36	2.41	19.26	
BC	15.72	0.11	25.44	—	47
ZIF-67	1258	0.59	—	48.30	48

a Crystallite sizes were calculated from the XRD data using the Scherrer equation.

### 3.3 Thermogravimetric and derivative thermogravimetric analysis (TG/DTG)

The thermal stability of ZIF-67, BC, and BCZIF-67 was assessed under a nitrogen atmosphere, with TG/DTG curves recorded from 20 °C to 800 °C, as shown in Fig. 3. The TG/DTG curve of ZIF-67 showed three stages of decomposition (Fig. 3a). The first mass loss observed between 250 °C and 301 °C (3.39%), was attributed to the loss of trapped guest molecules adsorbed in the pores of the ZIF-67 structure. This stage was followed by a significant mass loss of 35.25% between 450 °C and 560 °C, corresponding to the thermal breakdown of the 2-methylimidazolate organic linkers, resulting in framework collapse. An additional loss of 4.21% between 580 °C and 602 °C was attributed to the decomposition of residual carbonaceous matter, leaving a relatively high residual mass of approximately 57.15%,^37^ which was attributed to the carbon-based materials derived from 2-methylimidazole and cobalt-containing compounds such as Co and cobalt oxide.^38^

**Fig. 3:**
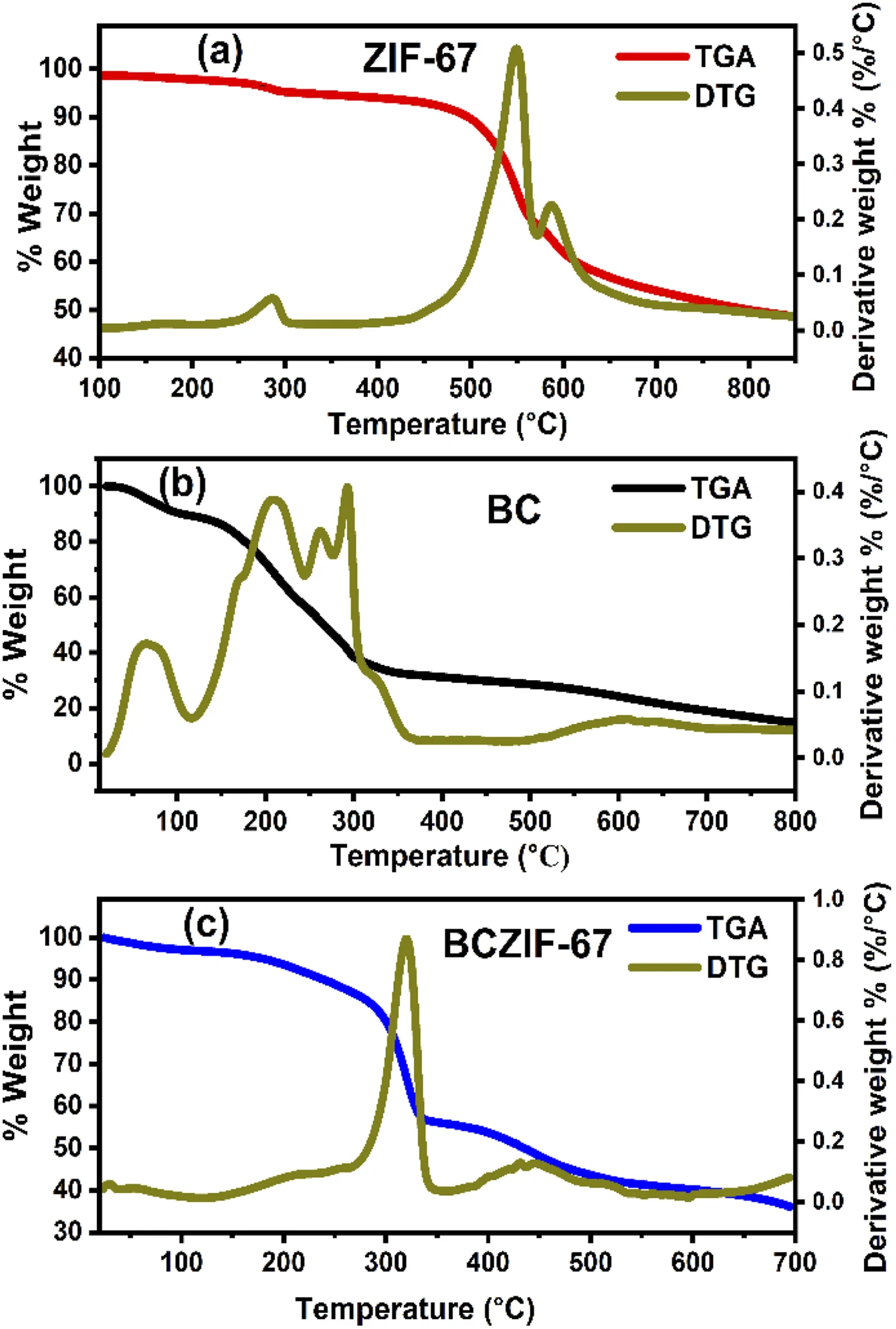
TG/DTG curves of (a) ZIF-67, (b) BC, and (c) BCZIF-67.

The TG/DTG curve for BC (Fig. 3b) revealed a typical multi-stage thermal decomposition profile of BC. An initial mass loss of 9.50% between 28 °C and 120 °C was attributed to the evaporation of residual moisture remaining from the drying process.^39^ This was followed by a major decomposition step of 60.65% between 150 °C and 360 °C, corresponding to the thermal decomposition of the cellulose backbone through depolymerization and cleavage of glycosidic linkages.^40^ A final mass loss of 4.11% at approximately 550 °C was associated with the gradual breakdown of residual char.^41^ The residual mass (∼25.74%) was attributed primarily to the formation of thermally stable carbonaceous char produced during dehydration and carbonization of the cellulose framework.^42^

The TG/DTG analysis of BCZIF-67 also revealed a multistep thermal decomposition profile (Fig. 3c). The composite exhibited a substantially smaller initial mass loss of 1.89% below 120 °C, indicating a lower moisture content, possibly because of the incorporation of ZIF-67 within the BC matrix. The second mass loss of 39.85%, observed between 270 °C and 350 °C, was attributed to the combined decomposition of the BC matrix and partial degradation of the imidazolate linkers in ZIF-67. A third mass loss of 14.85% between 450 °C and 600 °C was associated with further degradation of the ZIF-67 framework and residual carbonaceous material, leaving a final residue of 43.41%. The residual mass of BCZIF-67, intermediate between those of pristine BC and ZIF-67, is consistent with the coexistence of the thermally stable cobalt-containing inorganic species and the carbonaceous residue derived from bacterial cellulose, providing further evidence of composite formation.

### 3.4 N_2_ adsorption–desorption analysis

The textural properties of ZIF-67, BC, and BCZIF-67 are reported in Table 1. ZIF-67 showed a surface area of 1656 m^2^ g^−1^, a pore volume of 0.46 cm^3^ g^−1^, and a pore diameter of 0.83 nm, consistent with its microporous character.^43,44^ In contrast, BC exhibited a surface area of 15 m^2^ g^−1^, a pore volume of 0.06 cm^3^ g^−1^, and a pore diameter of 5.88 nm, consistent with its mesoporous character. Meanwhile, BCZIF-67 exhibited a surface area of 1305 m^2^ g^−1^, a pore volume of 0.36 cm^3^ g^−1^, and a pore diameter of 2.41 nm. Relative to pristine ZIF-67, BCZIF-67 showed a lower BET surface area (1305 *versus* 1656 m^2^ g^−1^) and pore volume (0.36 *versus* 0.46 cm^3^ g^−1^), consistent with partial blockage or reduced accessibility of ZIF-67 pores after composite formation.^45^ Nevertheless, the composite retained a high surface area and displayed additional mesoporous character associated with the BC network. The N_2_ adsorption–desorption isotherms and the corresponding pore distributions of ZIF-67, BC, and BCZIF-67 are shown in Fig. 4. ZIF-67 exhibited a type I isotherm, characteristic of microporous materials, whereas BC and BCZIF-67 showed type IV isotherms, typical of mesoporous materials. For BCZIF-67, the pore distribution showed a dominant peak at 2.41 nm with an additional tail extending to ∼10 nm, indicating the presence of both micro- and mesopores. These results indicate that BCZIF-67 integrates the microporosity of ZIF-67 with the mesoporous characteristics of BC, thereby producing a hierarchical pore system.^46^

**Fig. 4:**
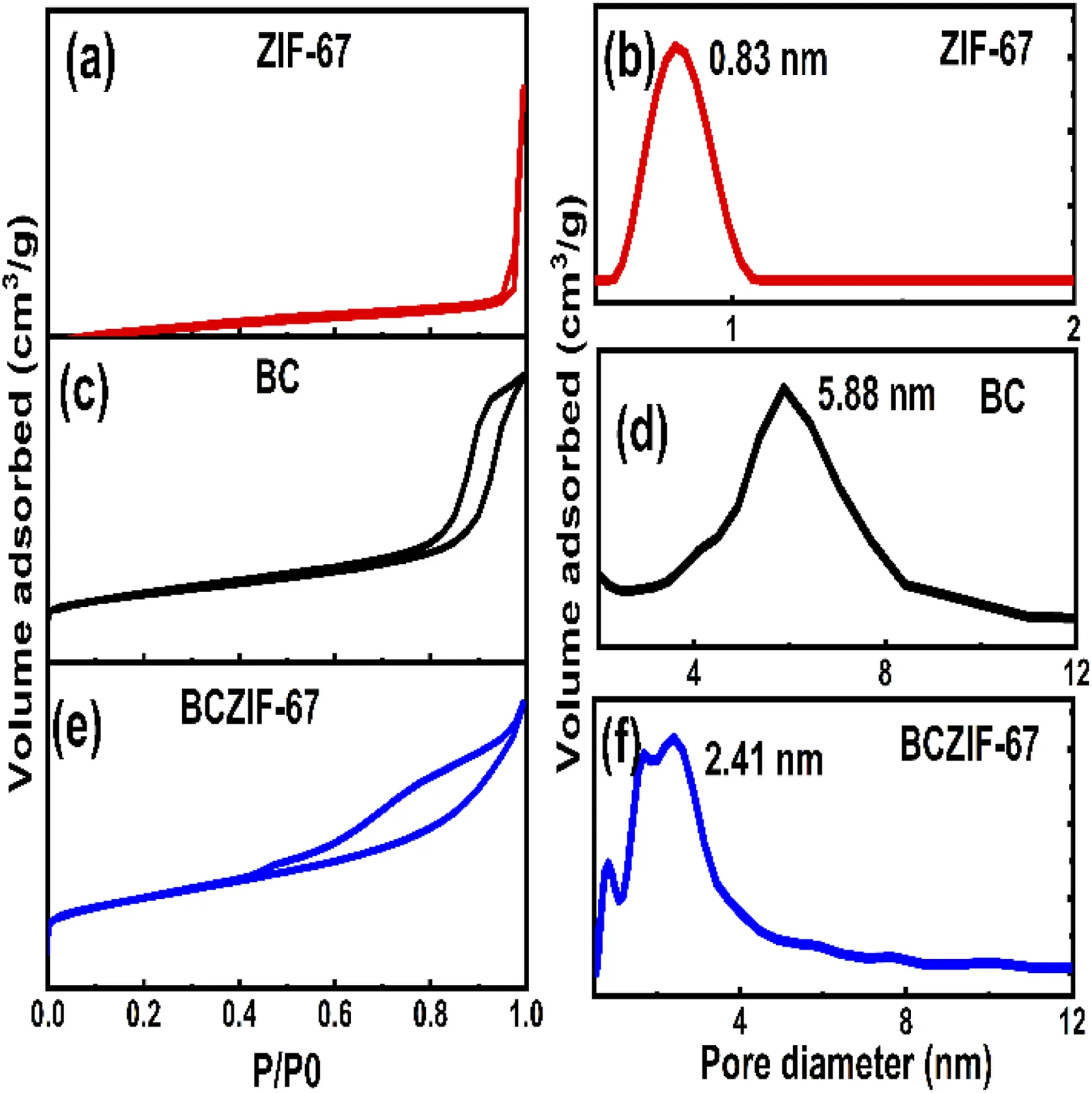
Nitrogen adsorption–desorption isotherms and corresponding pore size distributions of (a and b) ZIF-67, (c and d) BC, and (e and f) BCZIF-67.

### 3.5 Scanning electron microscopy (SEM) and energy-dispersive X-ray spectroscopy (EDS)

SEM was used to examine the surface morphologies of the synthesized materials. The SEM micrograph of ZIF-67 (Fig. 5a) showed rhombic dodecahedral particles with well-defined, smooth facets, consistent with the typical morphology of ZIF-67.^49^ The SEM micrograph of BC (Fig. 5b) showed a three-dimensional interwoven nanofibrous network with void spaces between the fibers, indicating a highly porous cellulose structure.^50^ The BCZIF-67 SEM micrograph (Fig. 5c) revealed irregular polyhedral-to-spherical particles anchored within the fibrous matrix. These observations suggest heterogeneous nucleation and growth of ZIF-67 crystals on the BC fibers, likely facilitated by coordination interactions between Co^2+^ ions and hydroxyl groups on the cellulose surface. Overall, the SEM results support the successful *in situ* growth of ZIF-67 on the BC matrix.

**Fig. 5:**
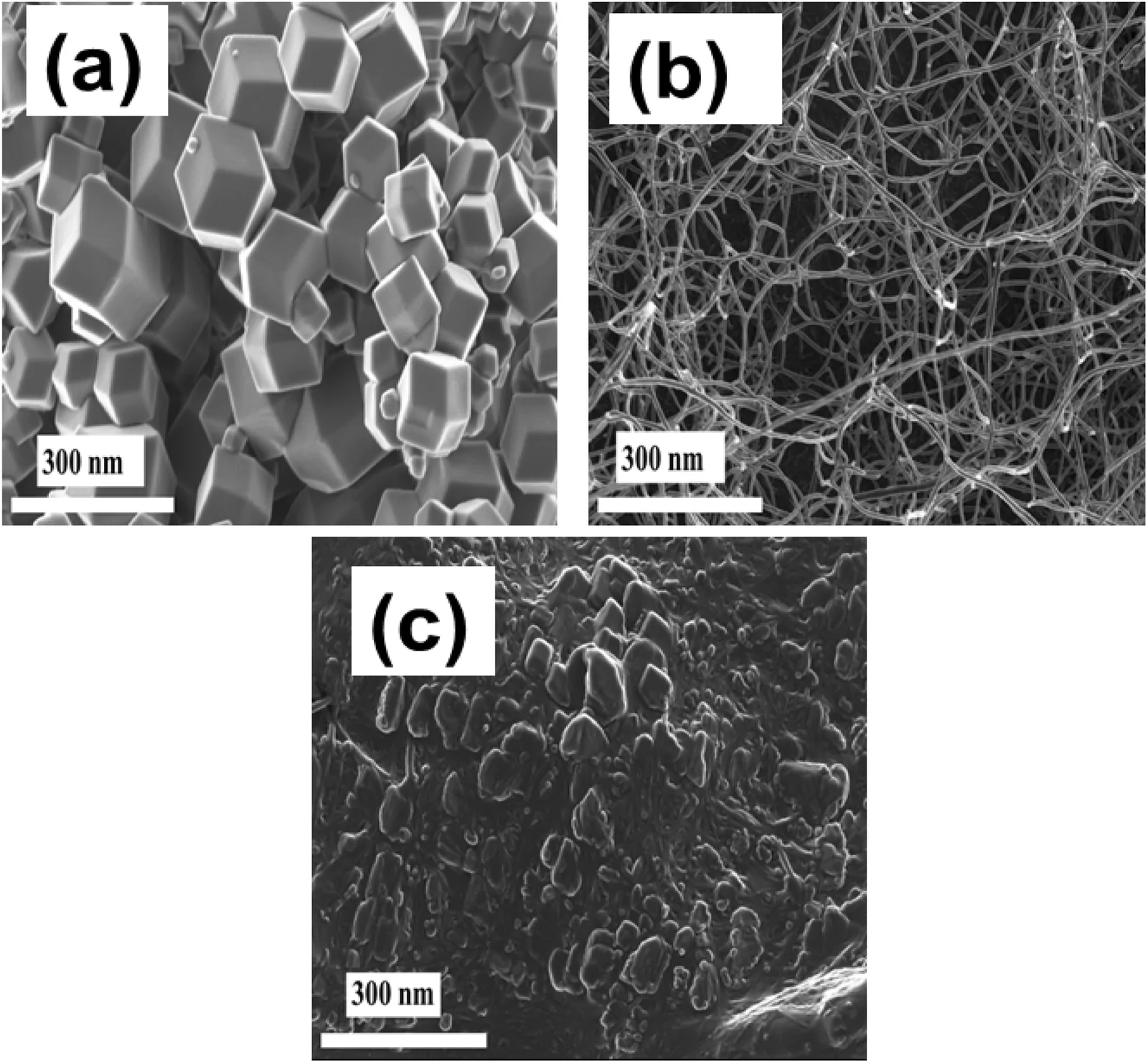
SEM micrographs of (a) ZIF-67, (b) BC, and (c) BCZIF-67.

To further examine the elemental composition of the synthesized BCZIF-67 composite, EDS analysis and elemental mapping were performed after SEM characterization. Fig. 6(a–d) presents the elemental maps of Co, O, C, and N, respectively. The corresponding overlaid elemental map (Fig. 6e) shows the local distribution of these elements within the selected region, supporting the incorporation of ZIF-67 into the BC matrix. Carbon and oxygen originate from both BC and ZIF-67, whereas nitrogen and cobalt arise from the imidazolate linker and cobalt centers of ZIF-67, respectively. The selected BCZIF-67 region contained Co (54.4 ± 0.2 wt%), O (10.7 ± 0.1 wt%), C (23.1 ± 0.1 wt%), and N (11.8 ± 0.3 wt%), as indicated by the EDS spectrum (Fig. 6f). Because EDS is a local, surface-selective technique, these values do not represent the absolute bulk loading of ZIF-67; however, the high local Co content supports substantial incorporation of ZIF-67 within the analysed region of the bacterial cellulose matrix.

**Fig. 6:**
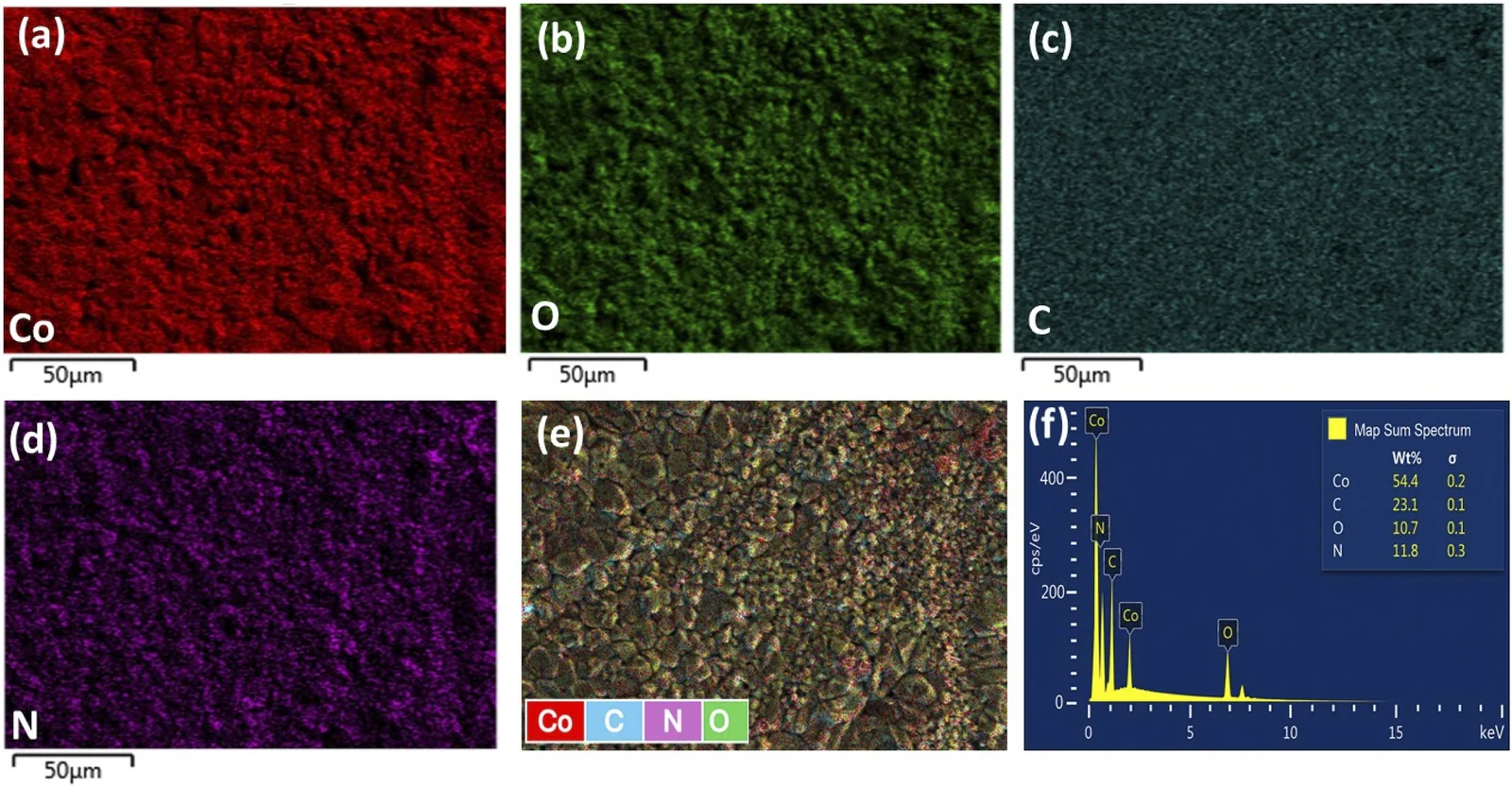
EDS mapping of BCZIF-67: (a) Co, (b) O, (c) C, (d) N, (e) overlaid elemental map of Co, O, C, N, and (f) EDS spectrum of BCZIF-67.

### 3.6 Optical absorption properties

The light absorption properties and optical band-gap energies of the prepared materials were evaluated using UV-visible diffuse reflectance spectroscopy (UV-vis DRS). As shown in Fig. 7a, ZIF-67 and BCZIF-67 exhibited characteristic absorption peaks in the ultraviolet region, attributed to ligand-to-metal charge transfer (LMCT) transitions.^51,52^ In contrast, BC displayed weak absorption in the UV region, consistent with its non-conjugated polysaccharide structure. Notably, BCZIF-67 also showed an additional absorption band in the visible region between 585 nm and 655 nm, corresponding to the ^4^A_2_(F) → ^4^T_1_(P) d–d electronic transition of tetrahedrally coordinated Co(ii) ions in the ZIF-67 framework.^53^ The presence of this visible-light absorption band indicates that BCZIF-67 exhibits broader light absorption compared with pristine ZIF-67.

**Fig. 7:**
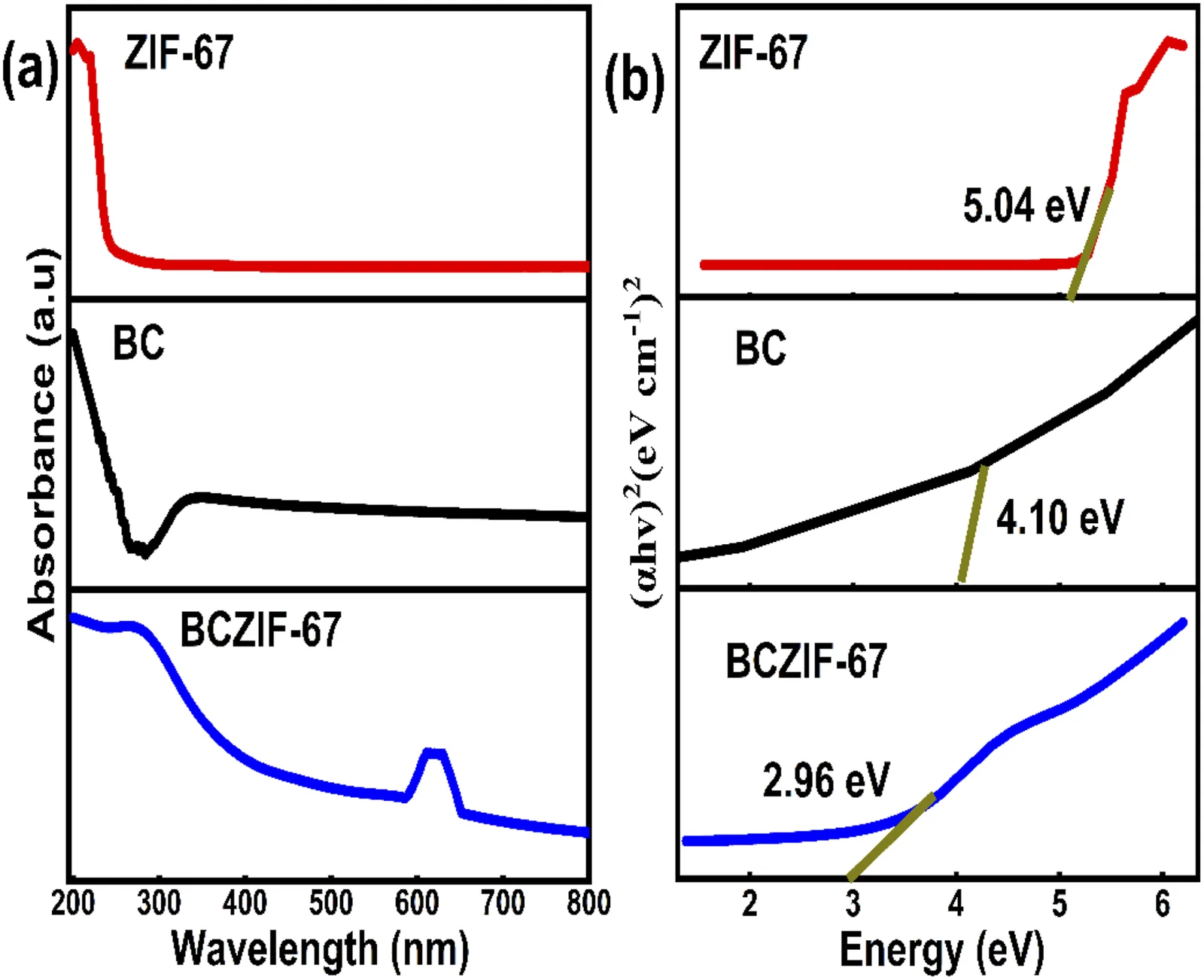
(a) UV-vis diffuse reflectance spectra of ZIF-67, BC, and BCZIF-67 and (b) corresponding direct transition Tauc plots used to estimate the apparent direct optical-gap energies.

The optical band gap (*E*_g_) was estimated from UV-vis DRS using the Kubelka–Munk transformation according to eqn (2).^54^2
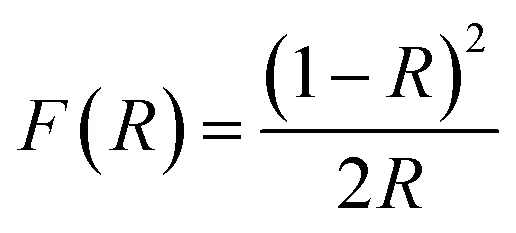
where, *F*(*R*) is the Kubelka–Munk function (dimensionless), which is proportional to the absorption coefficient for optically thick samples. *R* denotes the diffuse reflectance of the sample, expressed as a fraction. The optical band-gap energy (*E*_g_) was estimated using the Tauc relation (eqn (3)).^55^3
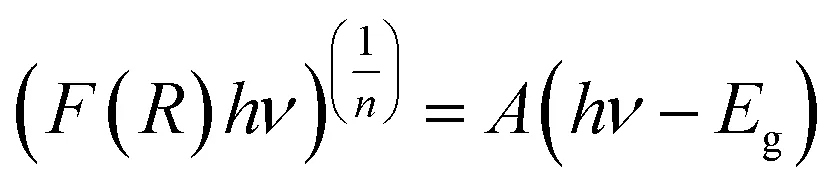
where *h* is Planck's constant, *ν* is the frequency of light, *A* is the proportionality constant, *E*_g_ is the optical band-gap energy, and *n* describes the type of electronic transition. Assuming a direct allowed transistion, *n* = 1/2, and the apparent optical-gap energies were obtained by extrapolating the linear regions of the [*F*(*R*)*hν*]^2^*vs. hν* plots to [*F*(*R*)*hν*]^2^ = 0.^56^

The direct-transition Tauc plots gave apparent optical-gap energies of 5.04, 4.10 and 2.96 eV for ZIF-67, BC and BCZIF-67, respectively. These values indicate that composite formation was associated with a substantial change in the optical response of the ZIF-67/BC system. The energy of the 365 nm irradiation used in the catalytic experiments is approximately 3.40 eV. This energy exceeds the apparent optical gap estimated for BCZIF-67 (2.96 eV), suggesting that photoexcitation of the composite is energetically feasible under the applied irradiation. In contrast, the photon energy is lower than the apparent optical gaps estimated for pristine ZIF-67 (5.04 eV) and BC (4.10 eV). Conventional band-to-band excitation of these two materials is therefore not expected at 365 nm on the basis of the present optical-gap estimates.

Accordingly, the reduction observed using pristine ZIF-67 should not be attributed unequivocally to direct band-to-band photoexcitation. Its activity may involve NaBH4 mediated surface catalytic processes, defect- or ligand-related sub-band-gap excitation, or other localized electronic transitions. Additional measurements, such as wavelength-dependent activity, photoluminescence spectroscopy and extended dark-reaction controls, would be required to distinguish these contributions.

The valence band (*V*_B_) and conduction band (*C*_B_) energies of the synthesized materials were estimated using the Mulliken electronegativity approach,^57^ as described in eqn (4) and (5):4
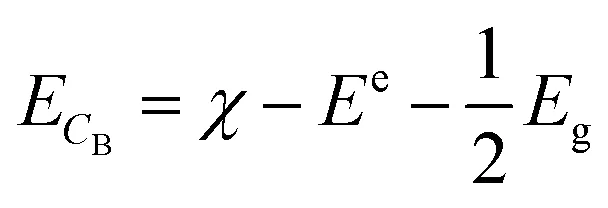

5 *E*_V_B__ = *E*_C_B__ + *E*_g_

where *E*_C_B__ and *E*_V_B__ denote the conduction band and valence band edge potentials, respectively; *E*^e^ is the free-electron energy on the hydrogen scale (∼4.50 eV); and *χ* is the absolute electronegativity of the semiconductor, estimated as the geometric mean of the absolute electronegativities of its constituent atoms.^58^

Based on this method, the band edge potentials of ZIF-67 were calculated as *E*_C_B__ = −1.96 V *vs.* NHE and *E*_V_B__ = 3.08 V *vs.* NHE. For BC, the corresponding values were *E*_C_B__ = 0.45 V *vs.* NHE and *E*_V_B__ = 4.85 V *vs.*NHE. The BCZIF-67 composite exhibited intermediate band positions with *E*_C_B__ = −0.13 V *vs.* NHE and *E*_V_B__ = 2.83 V *vs.* NHE. These values are consistent with the UV-vis DRS and Tauc analysis (Fig. 7), where the optical band-gap energy of BCZIF-67 (2.96 eV) was found to be narrower than that of pristine ZIF-67 (5.04 eV) and BC (4.10 eV). The shifts in the estimated conduction and valence band edges of BCZIF-67 may reflect interactions between ZIF-67 and the BC matrix and suggest altered charge-transfer behavior.

### 3.7 NaBH_4_-assisted photocatalytic reduction of NB

The photocatalytic reduction of NB to AN was monitored by UV-vis spectroscopy. As shown in Fig. 8a, the progressive decrease in absorbance at *λ* = 260 nm, corresponding to NB, was accompanied by the appearance of a new peak at *λ* = 210 nm, characteristic of AN. These spectral changes were consistent with NB depletion and the possible formation of AN in the presence of the catalysts.

**Fig. 8:**
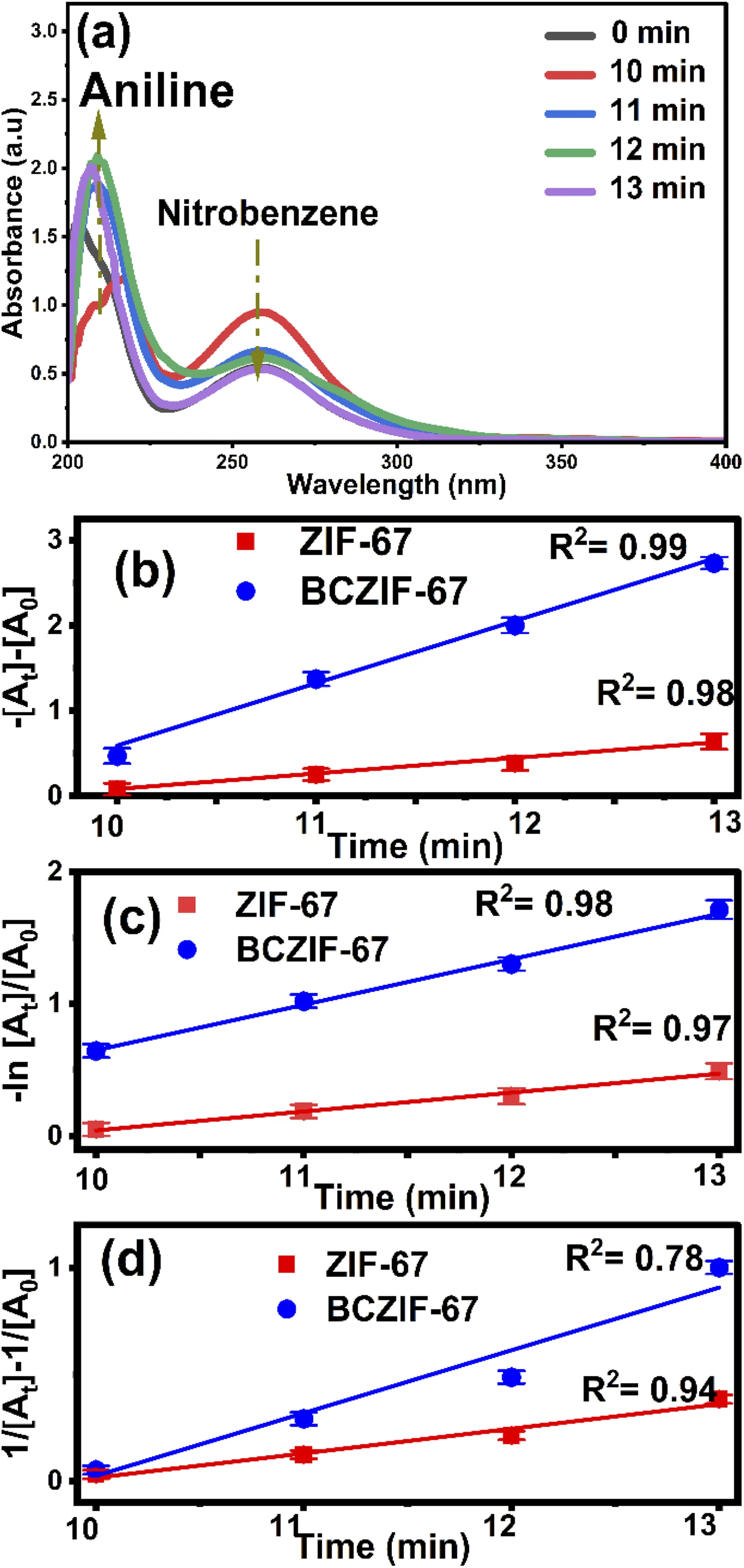
(a) UV-vis absorption spectral changes of NB in aqueous solution during NaBH_4_-assisted photocatalytic reduction over BCZIF-67 under UV irradiation. Kinetic plots for NB transformation over ZIF-67 and BCZIF-67 fitted to (b) zero-order, (c) first-order, and (d) second-order models.

To evaluate the reaction kinetics, the catalytic activities of ZIF-67 and BCZIF-67 were analyzed using zero-, first-, and second-order kinetic models. The general rate law is given by eqn (6); integration yields the linearized expressions presented in eqn (7)–(9) for zero-, first-, and second-order kinetics, respectively:6
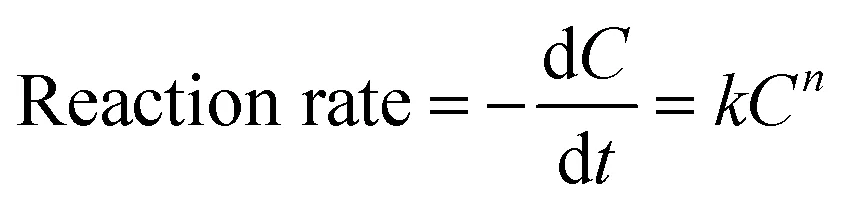

7 [*A*]_*t*_ − [*A*]_0_ = −*k*_app_^*t*^

8
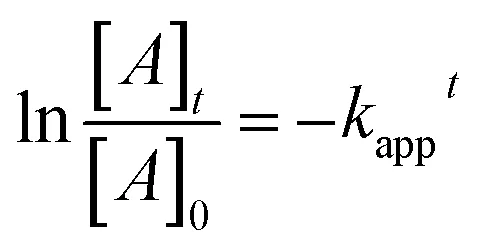
9
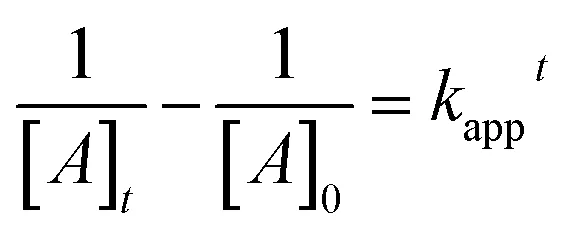
where *A*_*t*_ represent the absorbance of NB at time *t*, and *k*_app_ denotes the apparent absorbance-based reaction rate constant.

The data were plotted as shown in Fig. 8b–d. Each data set showed an approximately linear trend, with a slope corresponding to the rate constant. The coefficient of determination (*R*^2^) was used to assess the best fit. The results shown in Table 2 indicate that both zero- and first-order models fit the data well, with *R*^2^ of 0.99 and 0.98, respectively, whereas the second-order model showed a comparatively poorer fit. Although the zero-order model produced the highest *R*^2^ value, the difference between the zero- and first-order fits was small and did not, by itself, justify assigning a definitive zero-order mechanism. Moreover, because NaBH_4_ was present in large excess, pseudo-first-order behavior, commonly reported for NaBH_4_-assisted nitroaromatic reductions, cannot be excluded. Therefore, the observed kinetics are more appropriately described as exhibiting apparent zero-order behavior for this study rather than representing an intrinsic zero-order reaction mechanism. Whether this behavior reflects saturation of the catalyst surface or another rate-limiting process cannot be established from the present linearized fits. Initial rate measurements over a range of NB concentrations would be required to determine the reaction order and evaluate the contribution of surface site saturation.^59^ Under the zero-order linearization, BCZIF-67 exhibited a higher apparent absorbance based rate constant (0.69 ± 0.13 AU min^−1^) than pristine ZIF-67 (0.17 ± 0.05 AU min^−1^), indicating faster NB disappearance under the conditions investigated. This difference may be associated with improved dispersion of ZIF-67 particles within the porous BC network and greater accessibility of the catalytic sites.^60^

**Table 2:** Kinetic parameters for the photocatalytic reduction of NB over ZIF-67 and BCZIF-67 under UV irradiation, showing correlation coefficients (*R*^2^) and apparent absorbance-based rate constants (*k*_app_) for zero-, first-, and second-order models^a^

Reaction kinetics	*R* ^2^		Apparent rate constants, *k*_app_	
	ZIF-67	BCZIF-67	ZIF-67	BCZIF-67
Zero order	0.98	0.99	0.17 ± 0.05 (AU min^−1^)	0.69 ± 0.13 (AU min^−1^)
First order	0.97	0.98	0.13 ± 0.01 (min^−1^)	0.36 ± 0.02 (min^−1^)
Second order	0.94	0.78	0.10 ± 0.04 (AU^−1^ min^−1^)	0.36 ± 0.12 (AU^−1^ min^−1^)

a *k*_app_ denotes the apparent absorbance-based rate constant obtained directly from UV-vis. AU is the absorbance unit.

Control experiments performed without the catalyst or without NaBH_4_ showed negligible NB reduction (Fig. 9a). Under these conditions, the absorbance of NB remained essentially unchanged throughout the reaction period, indicating that little or no reduction occurred. This observation demonstrates that both the catalyst and the reducing agent are required to drive the reaction under the investigated conditions.

**Fig. 9:**
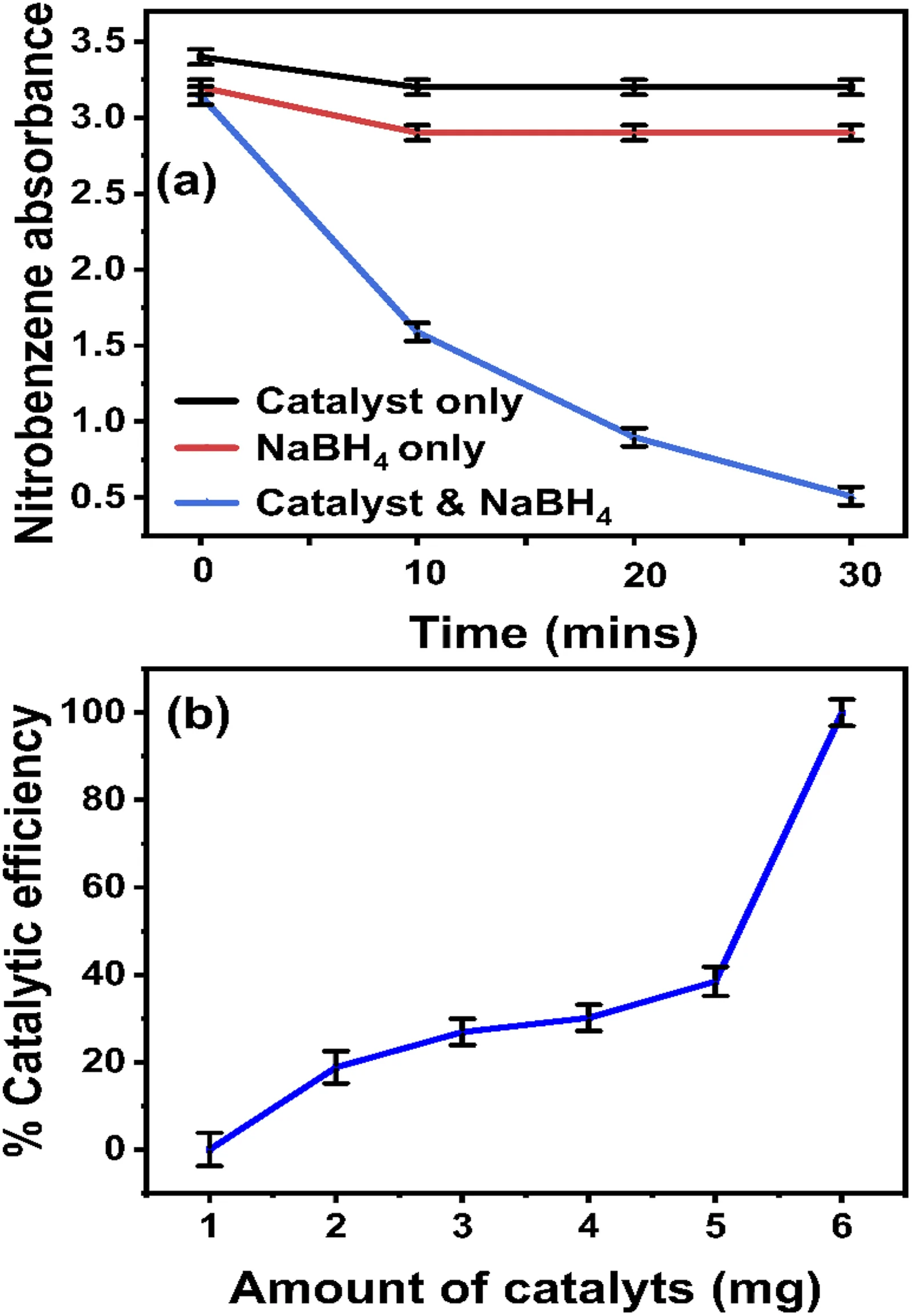
(a) Control experiment showing NB reduction in the absence of catalyst or NaBH_4_, (b) effect of BCZIF-67 catalyst dosage on the photoreduction of NB.

The photocatalytic performance was further evaluated by varying BCZIF-67 dosage from 1 to 6 mg (Fig. 9b). The apparent NB reduction efficiency increased with increasing catalyst loading and reached 100% with 6 mg of catalyst based on the decrease in NB absorbance at 260 nm. This dosage was therefore selected as the optimized condition for subsequent experiments. The results indicate that NB photoreduction is catalyst-dependent and that increasing the catalyst dosage provides a greater number of accessible active sites and increases the total population of photoexcited charge carriers, thereby accelerating the reduction of NB to AN.

The effect of NB concentration on the photocatalytic reduction process was investigated by systematically varying the NB concentration while maintaining a constant NaBH_4_ concentration and catalyst dosage, as shown in Fig. 10. At the relatively low NB concentration of 0.001 M, the photocatalyst achieved complete apparent NB reduction of NB. However, catalytic efficiency decreased substantially as the NB concentration increased. Specifically, at NB concentrations of 0.003 M and 0.005 M, the efficiency decreased to 27% and 15%, respectively.

**Fig. 10:**
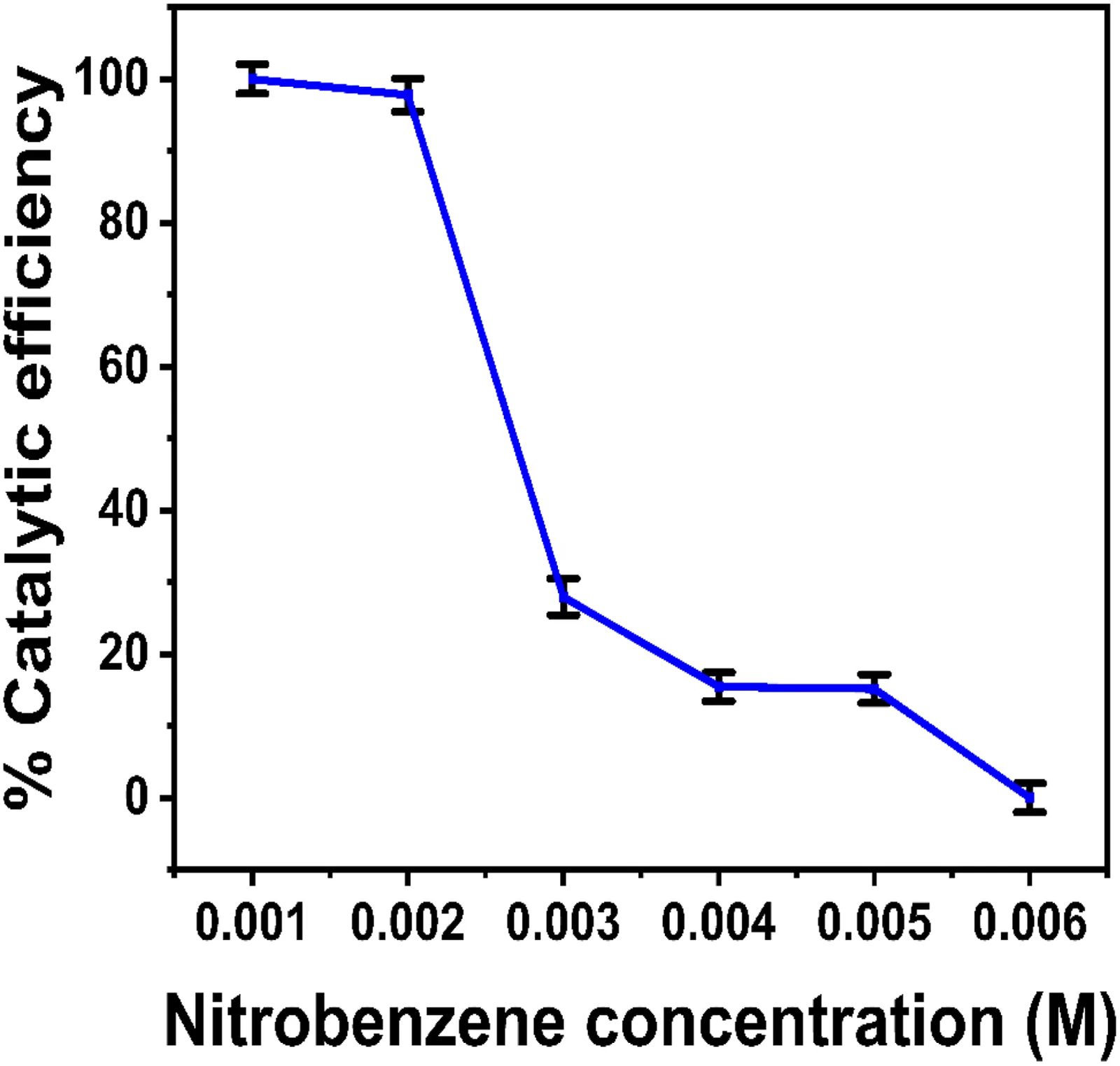
Effect of NB concentration on the photocatalytic reduction efficiency of BCZIF-67 under optimized conditions.

This pronounced decline in photocatalytic performance with increasing NB concentration may result from diminished light penetration into the reaction medium. As NB levels rise, the solution may become increasingly optically dense, restricting the light that can reach the photocatalyst's active surface.^61^ This attenuation of incident light may decrease the formation of electron–hole pairs, a crucial stage in the photocatalytic process, thereby hindering the efficient reduction of NB to AN.^62,63^ Based on these observations, an NB concentration of 0.001 M was identified as optimal for maintaining high photocatalytic activity and was therefore used consistently throughout this study.

The influence of the reducing agent was investigated by varying the NaBH_4_ concentration while maintaining constant NB and photocatalyst concentrations (Fig. 11). Increasing the NaBH_4_ concentration to 0.6 M substantially enhanced the catalytic activity. In contrast, decreasing the concentration to 0.4 M and 0.3 M reduced the catalytic efficiency to 79% and 20%, respectively. This trend indicates that higher NaBH_4_ concentrations provide a larger supply of reducing equivalents and promote electron transfer to adsorbed NB. The greater availability of reducing equivalents therefore facilitates the conversion of NB to AN and improved overall catalytic performance.

**Fig. 11:**
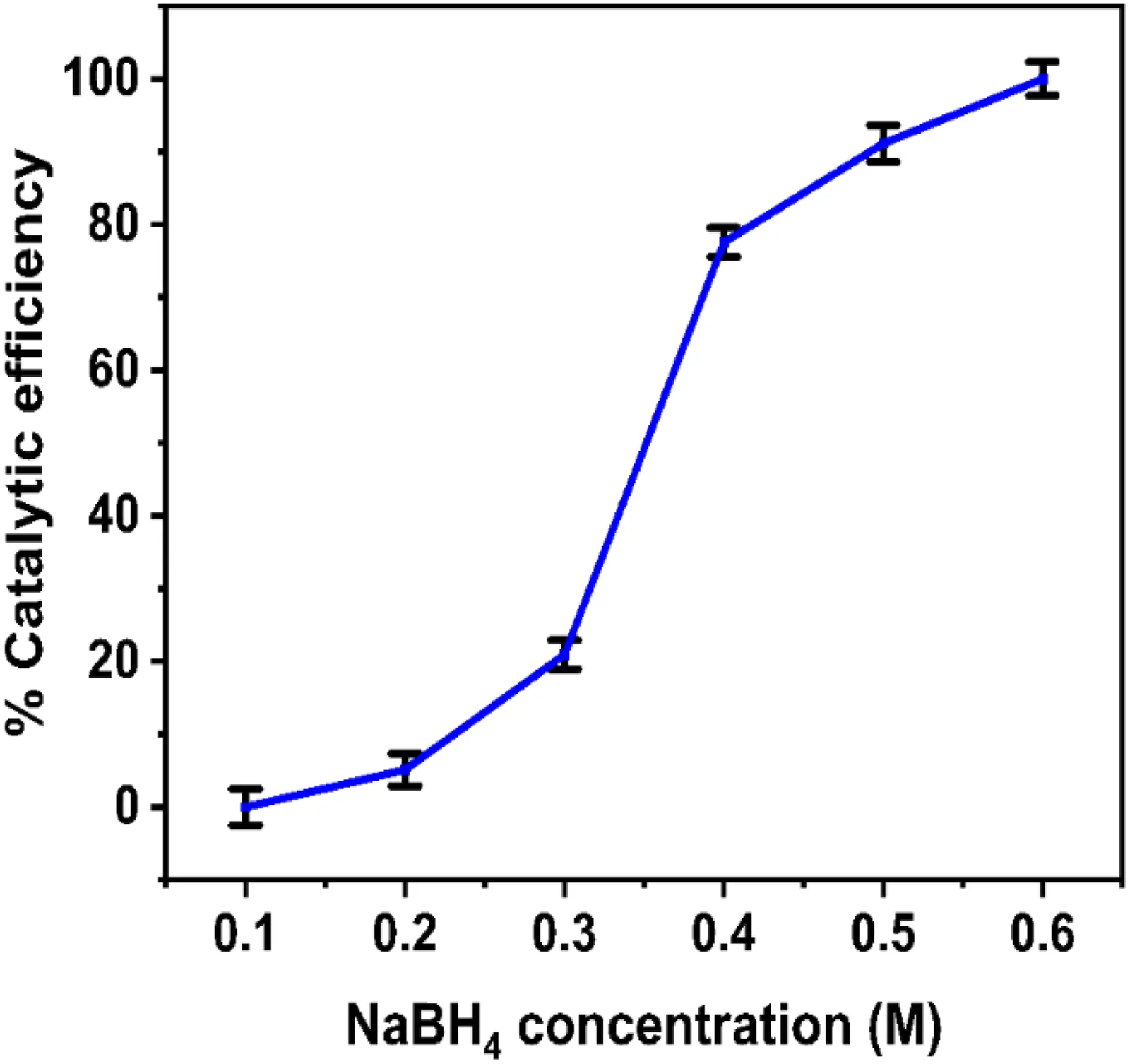
Effect of NaBH_4_ concentration on the photocatalytic reduction of NB over BCZIF-67 under optimized conditions.

The photocatalytic reduction efficiency of ZIF-67 and BCZIF-67 for NB was calculated using eqn (10),10
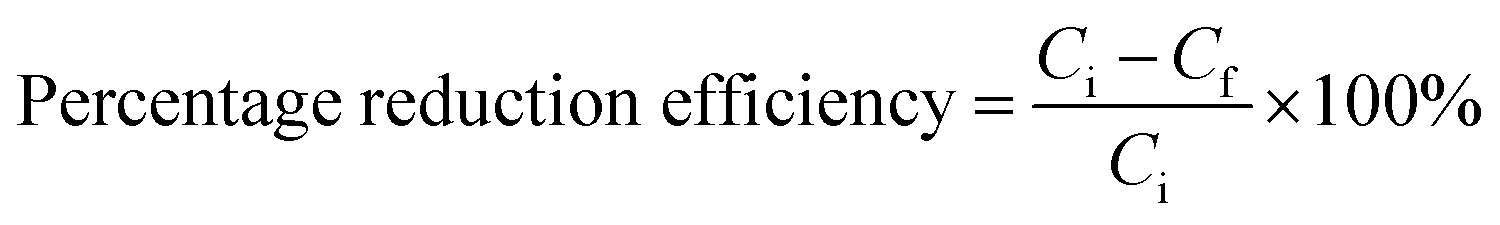
where *C*_i_ and *C*_f_ denote the initial and final concentrations of NB.

As shown in Fig. 12, BCZIF-67 achieved a maximum reduction efficiency of 100%, whereas ZIF-67 achieved only 40%.

**Fig. 12:**
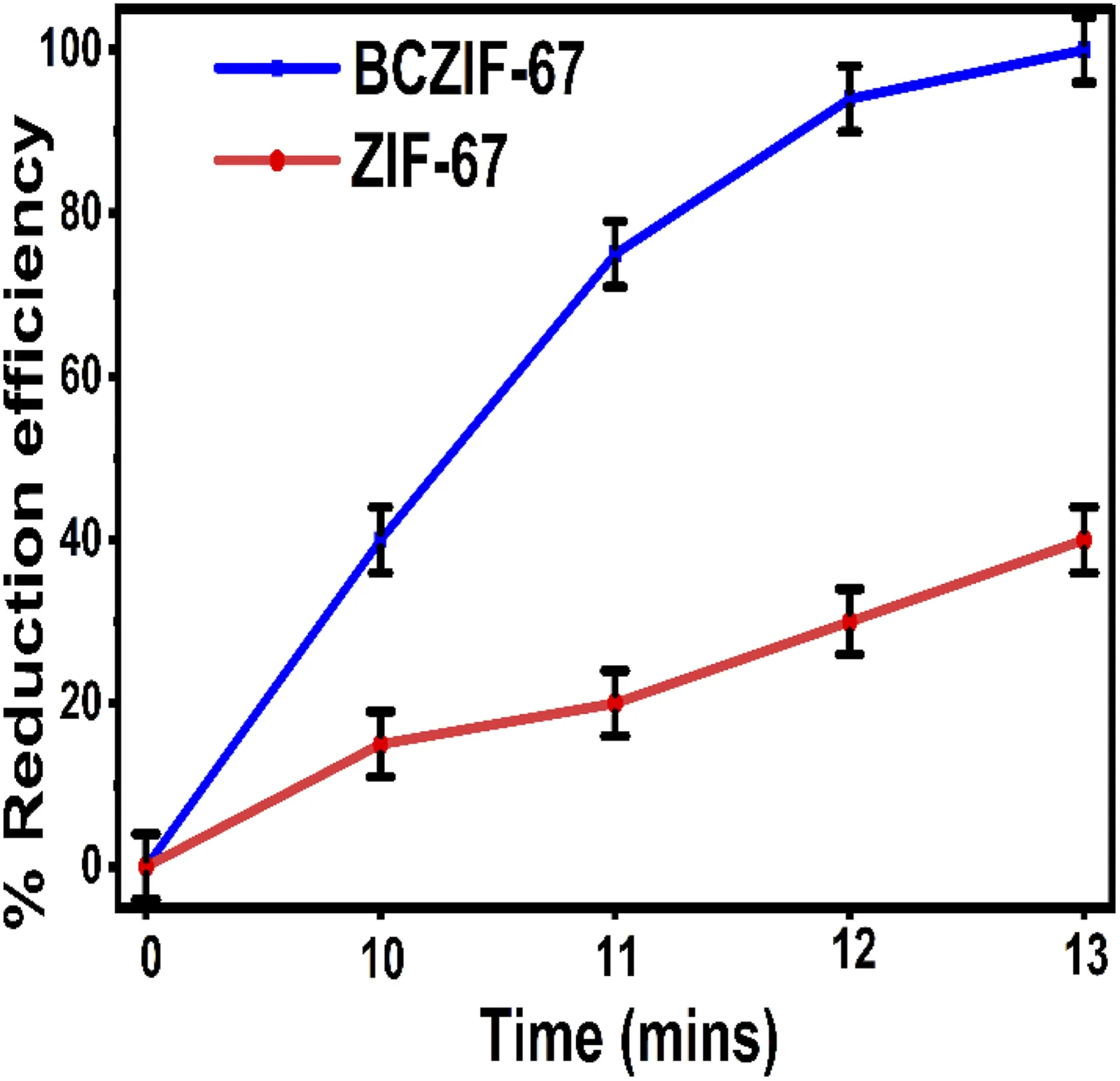
Photocatalytic reduction efficiency of ZIF-67 and BCZIF-67 for NB under optimized conditions.

To contextualize these results, Table 3 compares the photocatalytic performance of BCZIF-67 with that of previously reported ZIF-67, or MOF-based composites used to reduce NB. Under the optimized conditions used in this study, BCZIF-67 achieved 100% NB reduction within 13 min. This performance is comparable to those of previously reported systems, including CdS@NH_2_-MIL-125 (100%, in 1440 min), Cu-β-CD-MOF (79%, in 4 min), [Ni_3_(Ni-HTCPP)_2_(µ2 H_2_O)_2_(H_2_O)_4_(DMF)_2_] 2DMF (99%, in 90 min), and Cu@C (100% in 8 min).

**Table 3:** Comparative photocatalytic performance of BCZIF-67 and previously reported catalysts for photoreduction of NB and related pollutants

Catalyst	Efficiency (%)	Reaction time (min)	Light source	Target pollutant	Reference
CdS@NH_2_-MIL-125	100	1440	Visible light	NB	64
Cu-β-CD-MOF	79	4	Visible light	NB	65
[Ni_3_(Ni-HTCPP)_2_(µ2 H_2_O)_2_(H_2_O)_4_(DMF)_2_]·2DMF	99	90	Visible-light-assisted	NB	66
Cu@C	100	8	Visible light	NB	67
BCZIF-67	100	13	UV light	NB	This work

However, direct comparisons should be interpreted cautiously because the reported studies used different reaction conditions, pollutant concentrations, and catalyst dosages. The performance of BCZIF-67 is therefore more clearly demonstrated by its direct comparison with pristine ZIF-67 under identical experimental conditions: incorporation of BC increased the reduction efficiency from 40% to 100% and the apparent zero-order rate constant from 0.17 AU min^−1^ to 0.69 AU min^−1^.

The role of BC extends beyond that of a conventional physical support and can be understood through structure–property–performance relationships observed in this study. The abundant hydroxyl groups on the BC nanofibers provide anchoring sites for Co^2+^ ions during synthesis, promoting heterogeneous nucleation and *in situ* growth of ZIF-67 within the fibrous network. These interactions restrict uncontrolled crystal growth and aggregation, decreasing the average crystallite size from 63.36 nm for pristine ZIF-67 to 19.26 nm for BCZIF-67.^26^ Although incorporation of BC resulted in a decrease in BET surface area (from 1656 to 1305 m^2^ g^−1^) and pore volume (from 0.46 to 0.36 cm^3^ g^−1^), the composite retained a highly porous framework while providing improved dispersion of ZIF-67 particles within the BC network. This architecture may have increased the accessibility of catalytic sites and may have facilitated the diffusion of NB and NaBH_4_ to the active sites, contributing to enhanced photocatalytic performance.

The incorporation of BC also coincided with changes in the optical properties of the composite, including broader light absorption and a decrease in the estimated band-gap energy from 5.04 eV for pristine ZIF-67 to 2.96 eV for BCZIF-67. These results suggest that composite formation modifies the light responsive properties of ZIF-67. However, further spectroscopic and electrochemical investigations are required to establish the precise influence of the BCZIF-67 interface on charge separation and transfer. The enhanced catalytic performance of BCZIF-67 may therefore arise from the combined effects of structure-directed MOF nucleation, restricted crystal growth, hierarchical pore formation, improved accessibility of catalytic sites and modified optical properties.

Beyond the specific BCZIF-67 system, these results suggest that bacterial cellulose can function as a structure-directing scaffold rather than merely as a physical support. Its surface chemistry, MOF loading, and hierarchical pore architecture may be strategically controlled to regulate MOF growth, active site accessibility and reactant transport. These relationships offer potentially transferable design principles for developing MOF/BC composite catalysts, although their broader applicability must be validated across different MOF systems.

### 3.8 Stability and reusability

Catalyst recyclability is an important consideration in the development of heterogeneous photocatalytic technologies for wastewater treatment. In this study, the BCZIF-67 photocatalyst was recovered after each cycle, thoroughly washed with ethanol, and dried at room temperature for 24 hours before reuse. The catalyst was subjected to five consecutive cycles of NB reduction under UV light irradiation. These experiments provide an initial indication of catalyst reusability under the investigated conditions and should not be interpreted as a comprehensive assessment of long-term operational stability. As shown in Fig. 13a, BCZIF-67 maintained reduction efficiencies of 100%, 97%, 96%, and 90% during cycles 1–4, respectively, followed by a marked decrease to 45% in the fifth cycle.

**Fig. 13:**
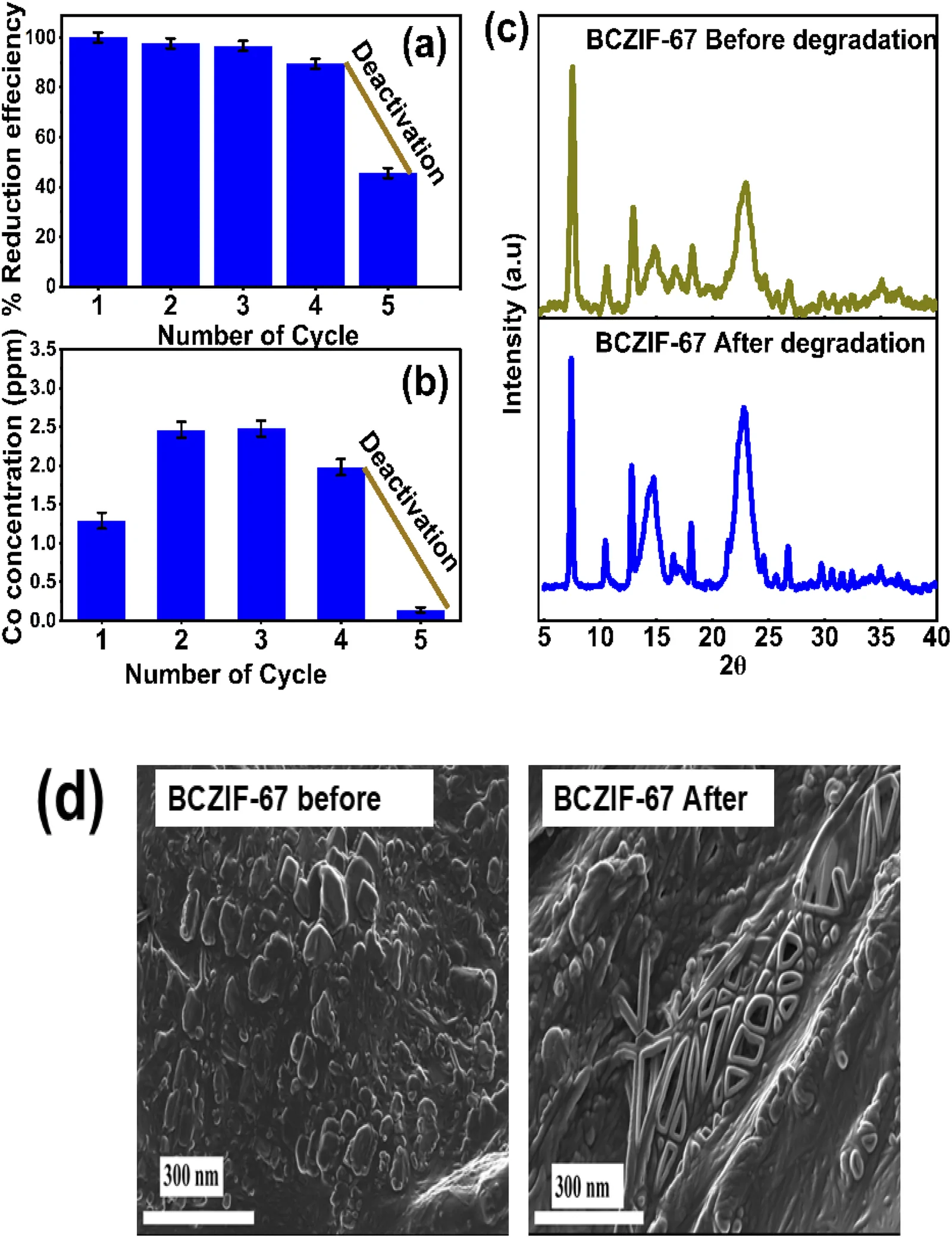
(a) Reusability of BCZIF-67 for NB photoreduction over multiple cycles; (b) cobalt concentration in solution during successive cycles; (c) XRD patterns, and (d) SEM images of BCZIF-67 before and after repeated NaBH_4_-assisted photocatalytic reactions.

The 10% decrease in photocatalytic efficiency over cycles 1–4 may be associated with catalyst loss during the recovery and washing, as well as catalyst leaching into the reaction solution. The pronounced decline in cycle 5 indicates substantial catalyst deactivation, potentially because of a reduction in the number of accessible active sites. These results show that BCZIF-67 retained high photocatalytic performance during the first four cycles, providing preliminary evidence of reusability. However, the marked activity loss in the fifth cycle highlights the need for extended recycling studies to assess the long-term durability and practical applicability of the catalyst.

To investigate whether catalyst leaching contributed to the observed decline, total Co concentrations in the reaction solutions were measured by MP-AES. The photocatalytic activity and corresponding Co (MP-AES) results for each reuse cycle are summarized in Table 4, whereas the catalyst reuse and Co leaching profiles are shown in Fig. 13a and b, respectively. As shown in Table 4 and Fig. 13b, the Co concentration increased over cycles 1–3, indicating progressive cobalt leaching from the BCZIF-67 composite during repeated use. The Co concentration then decreased during cycles 4 and 5 despite the continued decline in photocatalytic activity. This decrease may reflect depletion of readily leachable Co species or a reduction in the amount of catalyst remaining after repeated recovery. When considered together with Fig. 13a, the results suggest that partial loss or detachment of ZIF-67 nanoparticles from the surface and pores of the BC substrate may have contributed to the reduction in photocatalytic activity.

**Table 4:** Co leaching determined by MP-AES after each photocatalytic cycle of BCZIF-67

Cycle	Photocatalytic efficiency (%)	Total Co concentration (mg L^−1^)
1	100	1.29
2	97	2.46
3	96	2.48
4	90	1.98
5	45	0.14

To assess the structural stability of BCZIF-67 after five reuse cycles, XRD analysis was performed on the photocatalyst recovered after the fifth cycle. As shown in Fig. 13c, the diffraction pattern of the reused material was similar to that of pristine BCZIF-67, indicating that the composite largely retained its crystalline structure during NB photoreduction. Slight changes in peak intensity and broadening were nevertheless observed, suggesting minor structural disorder or reduced crystallinity, possibly because of repeated photocatalytic operation and partial catalyst leaching. SEM analysis was also performed on the catalyst recovered after the fifth reaction cycle (Fig. 13d). The micrographs showed slight surface roughening and localized structural distortion after repeated use, whereas the overall fibrous BC-supported ZIF-67 architecture remained intact. These minor structural changes, together with the detected catalyst leaching, are consistent with a partial loss of accessible active sites during repeated cycling and may explain the gradual decline in photocatalytic activity.

### 3.9 Proposed mechanism for light-assisted reduction of NB

A plausible mechanism for the light-assisted reduction of NB to AN over BCZIF-67 is illustrated in Scheme 1. The 365 nm irradiation corresponds to a photon energy of approximately 3.40 eV, which exceeds the apparent direct optical gap estimated for BCZIF-67 (2.96 eV). Photoexcitation of the composite is therefore energetically feasible under the applied irradiation. However, the proposed charge-transfer pathway remains tentative because charge-carrier separation and recombination were not measured directly. The estimated band-edge potentials of BCZIF-67 are approximately −0.13 V and +2.83 V *vs.* NHE for the conduction and valence bands, respectively.

**Scheme 1:**
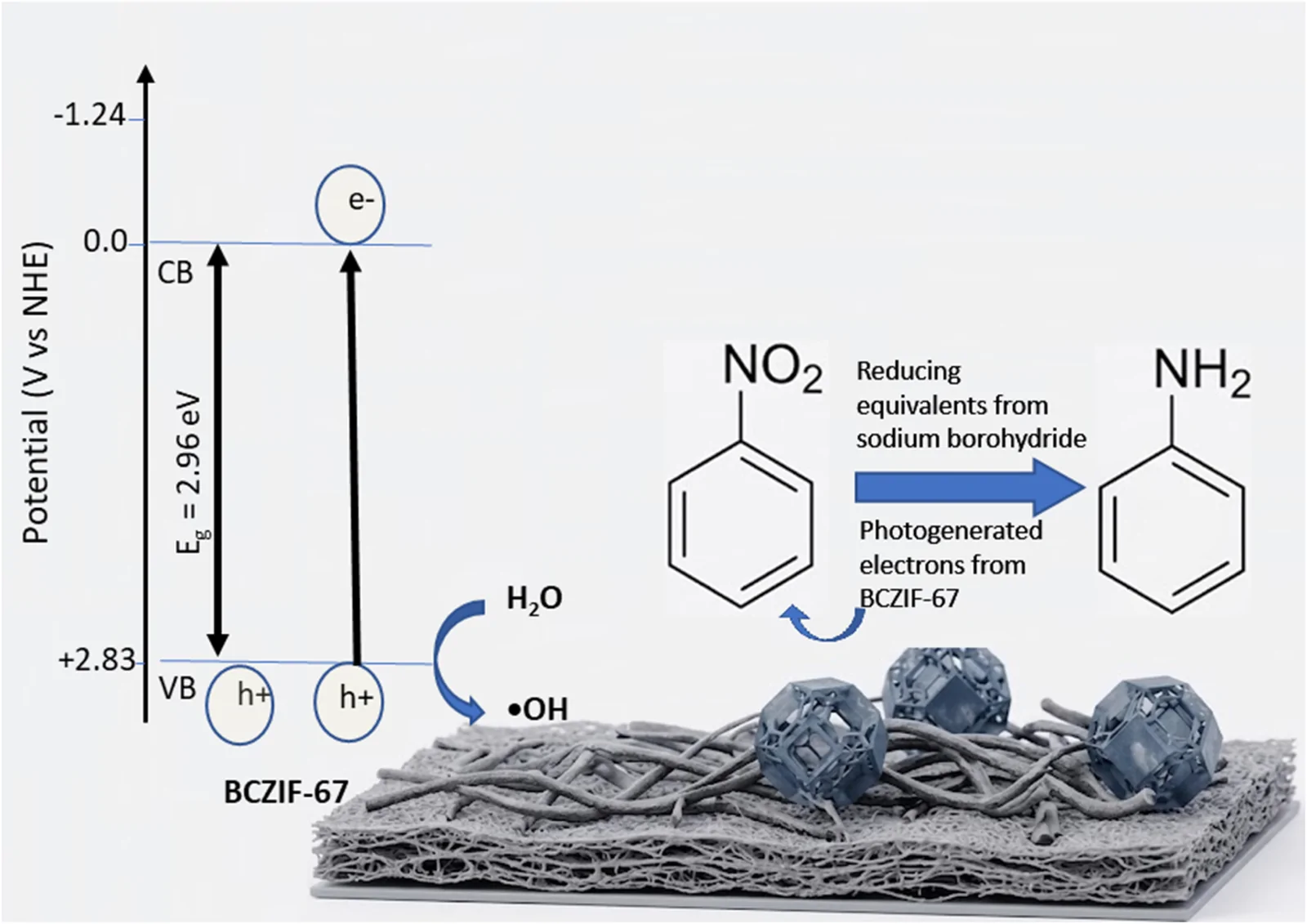
Proposed photocatalytic mechanism for the reduction of nitrobenzene over the BCZIF-67 composite under UV irradiation.

Within the proposed pathway, UV absorption by BCZIF-67 promotes electrons from the valence band to the conduction band and leaves holes in the valence band. Based on the calculated band-edge positions, the conduction band of BCZIF-67 is not sufficiently negative to reduce O_2_ to superoxide radicals (˙O_2_^−^). In contrast, the valence band is sufficiently positive to oxidize water or surface hydroxyl groups and may generate hydroxyl radicals.

Although hydroxyl radicals may participate in surface oxidation processes, NB reduction is proposed to depend primarily on the effective use of photogenerated electrons and reducing equivalents supplied by NaBH_4_. NaBH_4_ served as the stoichiometric reducing agent and supplied the reducing equivalents required for NB reduction. A plausible surface-mediated pathway involves transfer of reducing equivalents to adsorbed NB, followed by protonation steps involving the aqueous reaction medium. The reduction may proceed through nitrosobenzene and phenylhydroxylamine intermediates before aniline is formed. However, these intermediates, direct hydride transfer, and changes in charge-carrier lifetime were not measured experimentally; therefore, the proposed pathway remains tentative. The porous BC matrix may further facilitate this process by dispersing ZIF-67 active sites and promoting reactant transport through the composite. In the proposed BCZIF67 system, photogenerated electrons, along with equivalents donated by NaBH_4_, start the stepwise reduction process. This involves initially reducing nitrobenzene to nitrosobenzene, then to phenylhydroxylamine, and ultimately to aniline.

The addition of potassium iodide and ascorbic acid decreased the apparent NB reduction efficiency by approximately 63% and 49%, respectively (Fig. 14). These results demonstrate that both additives affected the reaction. However, potassium iodide and ascorbic acid may interact with the catalyst surface, NaBH_4_, reactive intermediates or photogenerated charge carriers and therefore cannot be considered selective probes under the present reaction conditions. Consequently, the observed inhibition cannot be assigned uniquely to photogenerated holes, hydroxyl radicals, superoxide radicals or a specific electron-transfer pathway. Additional selective trapping and spectroscopic measurements would be required to establish the identities and roles of the reactive species.

**Fig. 14:**
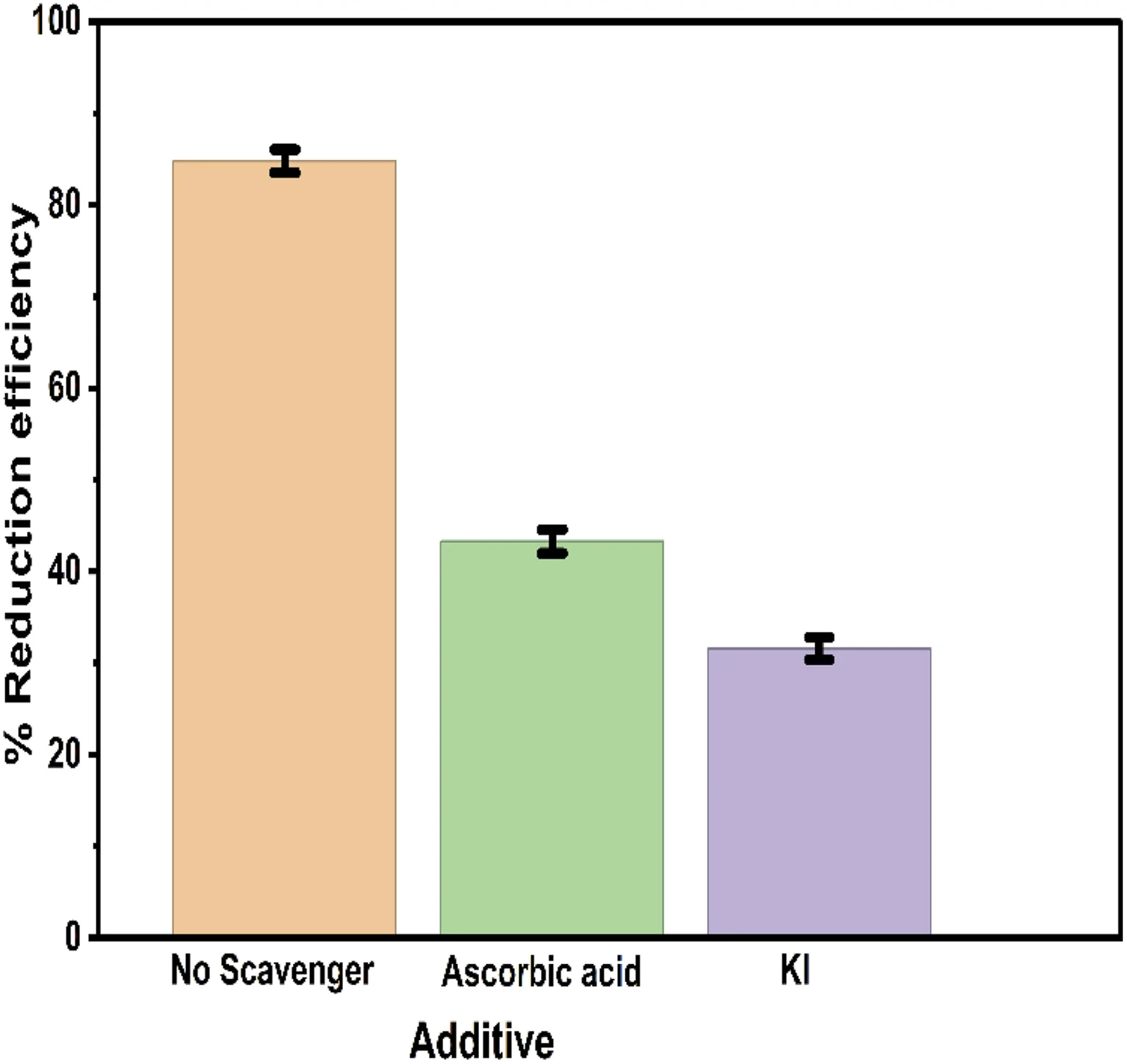
Comparison of NB reduction efficiencies in the absence of a scavenger and in the presence of ascorbic acid or potassium iodide.

## 4 Conclusion

This study demonstrates the successful development of a bio-based BCZIF-67 composite for the NaBH_4_ assisted catalytic reduction of nitrobenzene. Incorporating ZIF-67 into the BC matrix significantly enhanced the reduction efficiency, achieving 100% NB reduction within 13 minutes, compared with only 40% for pristine ZIF-67 under identical conditions. This enhanced performance is associated with the hierarchical porous structure of the BC support, improved dispersion and accessibility of the ZIF-67 active sites, broader light absorption, and potentially more efficient separation of photogenerated charge carriers. Kinetic analysis indicated an apparent zero-order response, suggesting that under the conditions examined, the reaction rate may be governed by the availability and saturation of active catalytic sites, consistent with heterogeneous catalytic systems. Reusability studies showed that BCZIF-67 retained relatively high activity over four consecutive cycles before significant deactivation occurred during the fifth cycle. XRD and SEM analyses indicated that the composite largely maintained its crystalline structure after repeated use. Band-edge estimates and scavenger-addition experiments provided a tentative basis for considering contributions from photoinduced charge-transfer processes and NaBH_4_ derived reducing equivalents. However, the identities and specific roles of the reactive species could not be established from the present experiments. These findings support a beneficial interaction between BC and ZIF-67 and highlight the potential of the MOF-cellulose composite as a sustainable material for light-assisted catalytic pollutant remediation. Future work will involve preparing composites with systematically varied ZIF-67 loadings to establish quantitative relationships between MOF loading, textural properties, optical characteristics and photocatalytic performance.

## Author contributions

Zandile Mhlwatika and Nolwazi Nombona contributed to the conception and design of the study. Material preparation and data collection were performed by Zandile Mhlwatika. Data analysis was performed by Zandile Mhlwatika, Samson Khene, and Nolwazi Nombona. Zandile Mhlwatika prepared the first draft of the manuscript and all authors commented on subsequent versions. All authors read and approved the final manuscript.

## Conflicts of interest

There are no conflicts to declare.

## Data Availability

The data supporting this study are available from the authors upon request and can also be accessed through the University of Pretoria Research Data Repository at 10.25403/UPresearchdata.33594490.
